# Synthesis, Structure, and Property of Tris(biphenyldiyl)yttrium(III) Tris(binaphthyldiyl)yttrium(III) and Tris(binaphthyldiyl)erbium(III) Complexes

**DOI:** 10.1021/acs.inorgchem.5c00532

**Published:** 2025-06-16

**Authors:** Masaki Hara, Gabriela Handzlik, Mirosław Arczyński, Takanori Iwasaki, Dawid Pinkowicz, Kyoko Nozaki

**Affiliations:** 1 Department of Chemistry and Biotechnology, Graduate School of Engineering, 13143 The University of Tokyo, 7-3-1 Hongo, Bunkyo-ku, Tokyo 113-8656, Japan; 2 Faculty of Chemistry, 37799 Jagiellonian University, Gronostajowa 2, Kraków 30-387, Poland

## Abstract

In the recent development of homoleptic σ-hydrocarbyl rare-earth metal complexes focusing on reactivity and catalytic activity, rare-earth metal complexes with a variety of alkyl ligands have been synthesized, and the principle for designing thermally stable σ-hydrocarbyl rare-earth metal complexes has been established. Nevertheless, there has been no report of homoleptic rare-earth metal complexes comprising only identical bidentate σ-hydrocarbyl ligands. Herein, we present homoleptic σ-hydrocarbyl rare-earth metal complexes possessing bidentate biaryldiyl ligands, tris­(biphenyl-2,2′-diyl)­yttrium­(III) complex **1**, tris­(1,1′-binaphthyl-2,2′-diyl)­yttrium­(III) complex **2**, and tris­(1,1′-binaphthyl-2,2′-diyl)­erbium­(III) complex **3**. Single-crystal X-ray crystallography revealed the trigonal prismatic geometry of **1**, along with the trigonal antiprismatic geometry of **2** and **3**, each constructed through three-centered two-electron bonds among the rare-earth metal, *ipso*-carbon, and lithium. The static and dynamic magnetic properties of **3** were investigated in the solid state, showing a slow magnetic relaxation typical of single-molecule magnets. The fitting of the magnetic behavior of **3** and the *ab initio* theoretical calculations indicated that the magnetic relaxation of **3** occurs primarily through a Raman relaxation process.

## Graphical abstract

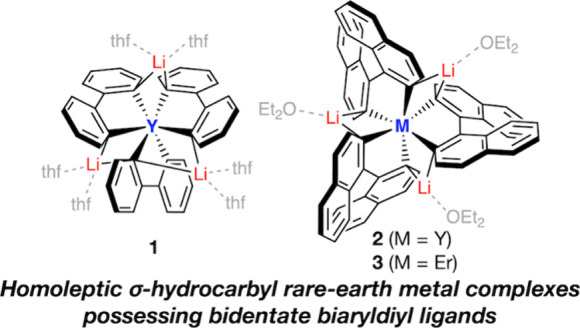

## Introduction

Homoleptic hydrocarbyl rare-earth metal complexes have experienced significant growth in the last half century, driven particularly by their importance in Ziegler-type polymerization catalysis, possessing coordinatively unsaturated early transition metals.^1^ The high reactivity of these complexes has also allowed their use as precursors for the synthesis of various heteroleptic derivatives, providing the basis for the development of rare-earth-mediated catalysis.^2^

The first well-defined archetypal examples of homoleptic rare-earth metal hydrocarbyls were dominated by the π-complexes sandwiched between cyclopentadienyl ligands, following the discovery of ferrocene in the 1950s.^3^ Wilkinson reported the tricyclopentadienyl complexes of Sc, Y, and some lanthanoids in 1954,^4^ followed by abundant reports due to their ease of substitution and modification.^5^ Subsequently, the first σ-bonded rare-earth metal aryl compound, triphenylscandium, appeared in 1968.^6^ After the breakthrough independently achieved in the 1970s by Lappert et al.^7^ and Wilkinson et al.,^8^ describing the introduction of bulky alkyl groups to obtain kinetically stable organo-transition metal alkyls, trimethylsilylmethyl groups have been widely applied to rare-earth metals due to the absence of β-hydrogen, suppressing decomposition via β-hydride elimination.^9^ Furthermore, in the early 1980s, permethylated rare-earth metal compounds [MMe_6_]^3–^ were found to be obtained in a thermally stable condition supported by the contact of Li^+^ ions.^10^ To date, there have been abundant reports of homoleptic rare-earth metal σ-hydrocarbyl complexes, typically with methyl, ethyl, ^*t*^butyl, neopentyl, trimethylsilylmethyl, alkynyl, phenyl, and benzyl groups.^2^

In attempts to develop homoleptic σ-hydrocarbyl complexes, introducing bulky alkyl groups without β-hydrogens has not always been effective in enhancing the thermal stability of rare-earth metal complexes,^11^ leading to challenges in crystallographic structure determination in some cases. One reasonable idea to increase the stability of homoleptic σ-hydrocarbyl complexes is to extend the monodentate ligands to bidentate ligands to form a rigid structure stabilized by chelation, as M­(*o*-Me_2_N–C_6_H_4_CH_2_)_3_ (M = Y, La) has been reported to show high thermal stability even at 75 °C in toluene, being stabilized by intramolecular coordination of the *ortho*-NMe_2_ substituent.^12^ When it comes to bidentate σ-hydrocarbyl ligands, monoanionic Ce and La complexes possessing a biphenyl-2,2′-diyl ligand (bph) along with two cyclopentadienyl ligands have been reported;^13^ however, there has been no report of homoleptic examples comprising only identical bidentate σ-hydrocarbyl ligands, leaving an unexplored field of investigation involving this intriguing model of rare-earth metal complexes. Among the early transition metals, homoleptic complexes consisting solely of biphenyl-2,2′-diyl ligands are known for Cr, V, and Zr (**A**–**C** in Scheme 1).^14^ In addition, we previously reported that a π-extended analog having solely 1,1′-binaphthyl-2,2′-diyl ligands (bnph) is also accessible for Rh and Ir (**D** and **E** in Scheme 1).^15^

**Scheme 1:**
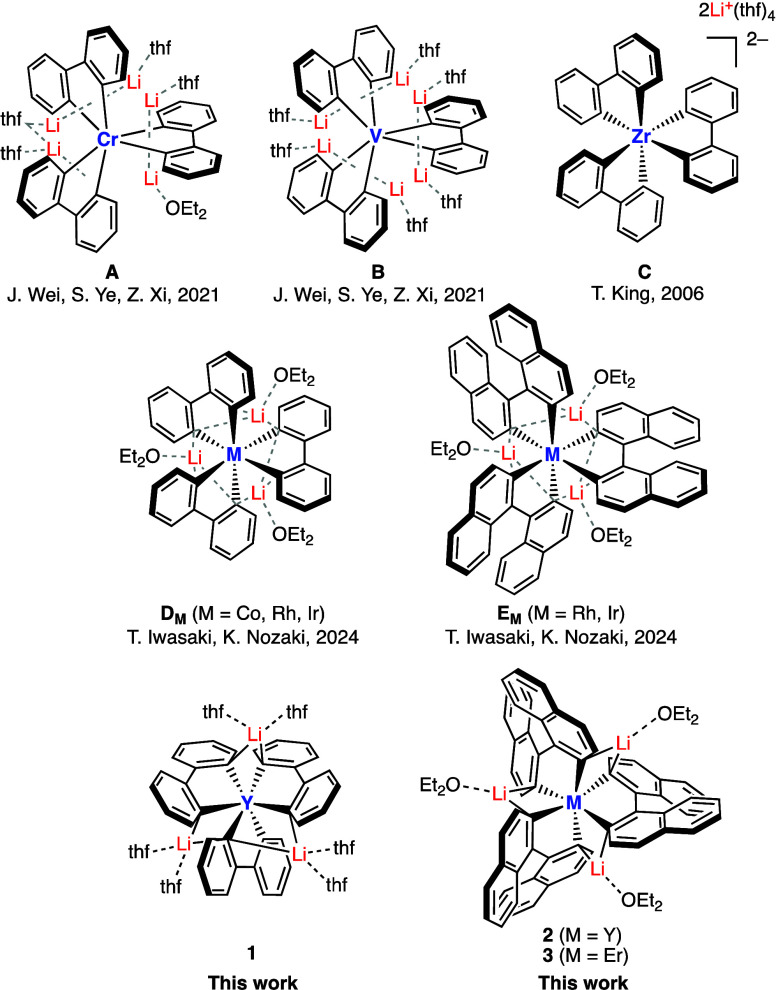
Homoleptic Tris­(biaryldiyl) Transition Metal Complexes

In this report, we present a new series of homoleptic rare-earth metal complexes having only three identical bidentate σ-hydrocarbyl ligands: tris­(biphenyl-2,2′-diyl)­yttrium­(III) complex (Li_3_Y­(bph)_3_ (**1**)), and the π-extended analogs, tris­(1,1′-binaphthyl-2,2′-diyl)­yttrium­(III) and tris­(1,1′-binaphthyl-2,2′-diyl)­erbium­(III) complexes (Li_3_Y­(bnph)_3_ (**2**) and Li_3_Er­(bnph)_3_ (**3**)) (Scheme 1). These complexes showed sufficient thermal stability to be isolated, and their structure was determined by X-ray crystallography. Complexes **2** and **3** were obtained as a single diastereomer in racemic form, each consisting of a pair of mirror isomers arising from the axial chirality of the binaphthyl skeletons. The Er analog **3** was characterized by means of direct current (DC) and alternating current (AC) magnetic measurements and shows a field-induced slow magnetic relaxation typical for single-molecule magnets.^16^

## Results and Discussion

### Synthesis

Complexes **1** and **2** were synthesized by treating YCl_3_ (**4**) with corresponding dilithio reagents, 2,2′-dilithiobiphenyl (**5**) and 2,2′-dilithio-1,1′-binaphthyl (**6**), respectively (Scheme 2). Complex **1** was formed within 10 min by treating **4** with 3.5 equiv of **5** in THF. The mixture was suspended in excess hexane, the precipitate was filtered off, and the remaining solution was concentrated under reduced pressure until a small amount of the mother liquor remained, yielding colorless needle-like crystals of **1** in 12% yield (Scheme 2a). Complex **2** was gradually precipitated from a mixed solution of **4** and 3.5 equiv of racemic **6** in THF over several days. The rough crystals of **2** were washed with Et_2_O, followed by recrystallization employing slow diffusion of pentane into a benzene solution of **2** to give colorless block crystals of **2** in 27% yield (Scheme 2b). The same complex **3** with Er as the metal center was produced within 1 h through a reaction between ErCl_3_ (**7**) and racemic **6** in THF. Further purification by recrystallization from Et_2_O afforded colorless block crystals of **3** in 15% yield (Scheme 2c). The low yields of complexes **1** and **3** are due to their high solubility in organic solvents, which leads to low recrystallization efficiency, whereas complex **2** has a lower solubility and thus precipitates during the reaction. The low yield of **2** probably originates from the inherently low yield of the reaction between YCl_3_ (**4**) and **6.**

**Scheme 2:**
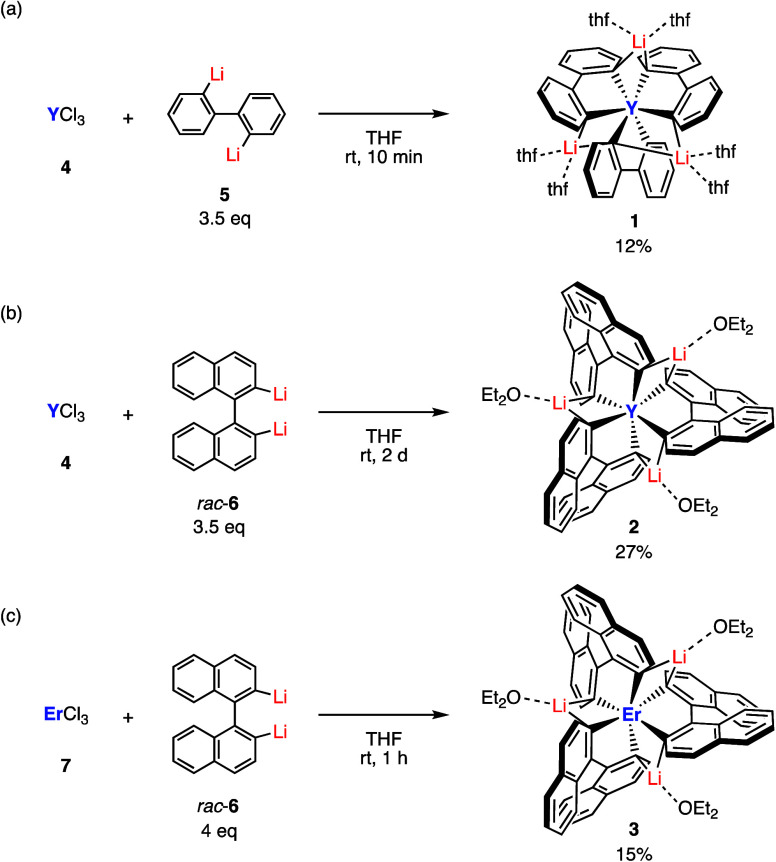
Synthesis of Tris­(biphenyldiyl)­yttrium­(III) Complex **1**, Tris­(binaphthyldiyl)­yttrium­(III) Complex **2**, and Tris­(binaphthyldiyl)­erbium­(III) Complex **3**

### Spectroscopic Characterization

^1^H and ^13^C NMR spectra of complex **1** in nondeuterated diethyl ether showed only one set of *C*_2_-symmetric biphenyl-2,2′-diyl moiety in the aromatic region (Figures S4 and S5). Complex **1** exhibited a doublet signal at 185.5 ppm (^1^ *J*_Y–C_ = 23.7 Hz) in its ^13^C NMR spectrum corresponding to the *ipso*-carbon (C_*ipso*_) atoms. The ^1^ *J*_Y–C_ coupling constant is considerably smaller than those reported for homoleptic trimethylsilylmethyl complexes; [Y­(CH_2_SiMe_3_)_3_(thf)_2_] (^1^ *J*_Y–C_ = 35.4 Hz, THF-*d*_8_),^17^ [Y­(CH_2_SiMe_3_)_2_(thf)_4_]­BPh_4_ (^1^ *J*_Y–C_ = 41.2 Hz, THF-*d*_8_)^18^ and [Y­(CH_2_SiMe_3_)­(thf)_4_]­(BPh_4_)_2_ (^1^ *J*_Y–C_ = 44.9 Hz, pyr-*d*_5_),^18^ indicating the relatively weakened Y–C bonds in **1** due to the formation of three-center two-electron (3c–2e) bonds among the rare-earth metal, *ipso*-carbon and lithium (*vide infra*). The NMR analysis of complex **2** was hampered by its low solubility and stability in organic solvents. Complex **2** barely dissolved in benzene, allowing us to conduct ^1^H NMR measurements and observe only one set of *C*_2_-symmetric 1,1′-binaphthyl-2,2′-diyl moiety in the aromatic region, although we should note that contamination of 1,1′-binaphthyl via the partial decomposition of **2** was observed (Figure S7).

### Structural Characterization

Single-crystal X-ray crystallography revealed an approximately trigonal prismatic structure of complex **1** with three metalla-fluorene skeletons fused at a Y atom, where three Li^+^ ions are in contact with the anionic counterpart (Figure 1). In the unit cell, three molecules of **1** with slightly different geometries (**1**_**A**_, **1**_**B**_, and **1**_**C**_) are observed (Table 1). In molecules **1**_**A**_ and **1**_**B**_, each Li^+^ ion is coordinated by two of the *ipso*-carbon atoms (C1 and C2 for Li1, C5 and C6 for Li2, and C1 and C3 for Li3), being sandwiched between aryl moieties. While Li1 and Li3 both contact C1, Li2 independently contacts C5 and C6, which have no interaction with Li1 and Li3, resulting in asymmetric structures of **1**_**A**_ and **1**_**B**_ with no rotational symmetry axis. The *ipso*-carbon C1, sandwiched between two Li^+^ ions (Li1 and Li3), has a significantly longer Y–C bond (2.631(5) Å (**1**_**A**_) and 2.612(5) Å (**1**_**B**_)), whereas the *ipso*-carbon C4, which has no interaction with any Li^+^ ions, shows significantly shorter Y–C bond (2.485(6) Å (**1**_**A**_) and 2.462(6) Å (**1**_**B**_)), compared to other Y–C bonds (2.547(5)–2.576(5) Å (**1**_**A**_) and 2.530(6)–2.580(6) Å (**1**_**B**_)). The biphenyldiyl moiety, including *ipso*-carbon atoms C1 and C4, has a nearly planar structure with torsion angles of 5.1(7)° (**1**_**A**_) and 3.2(7)° (**1**_**B**_). On the other hand, the other two biphenyldiyl moieties are slightly twisted with torsion angles of 29.1(7)–30.6(7)° (**1**_**A**_) and 32.2(8)–34.3(9)° (**1**_**B**_). In molecule **1**_**C**_, the molecular structure is almost the same as those of **1**_**A**_ and **1**_**B**_, except for a considerably longer Li3–C1 bond (2.63(1) Å). Along with the weakening of the Li3–C1 interaction, **1**_**C**_ has a relatively short Y–C1 bond (2.588(5) Å), as well as increased torsion angle within the biphenyldiyl moiety including C1 and C4 (9.3(7)°), compared to **1**_**A**_ and **1**_**B**_.

**Figure 1:**
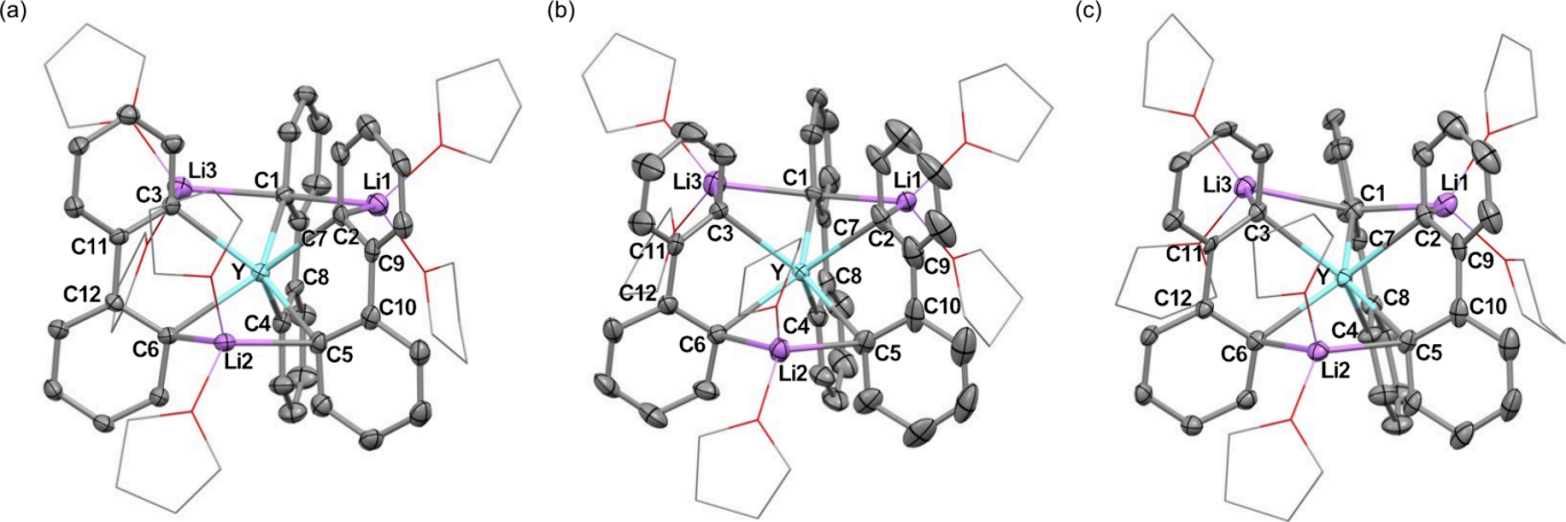
ORTEP drawing of (a) **1**_**A**_, (b) **1**_**B**_, and (c) **1**_**C**_ with thermal ellipsoids at the 50% probability level, except for coordinating THF molecules, which are shown in the wireframe model. All hydrogen atoms, noncoordinating solvent molecules, and disordered molecules are omitted for clarity.

**Table 1:** Selected Bond Lengths (Å) and Torsion Angles (deg) of the Tris­(biphenyldiyl)­yttrium­(III) Complex

	**1_A_**	**1_B_**	**1_C_**
Y–C1	2.631(5)	2.612(5)	2.588(5)
Y–C2	2.576(5)	2.575(6)	2.564(6)
Y–C3	2.547(5)	2.580(6)	2.602(5)
Y–C4	2.485(6)	2.462(6)	2.498(5)
Y–C5	2.556(5)	2.559(5)	2.561(4)
Y–C6	2.571(5)	2.530(6)	2.550(6)
Li1–C1	2.38(1)	2.35(1)	2.31(1)
Li1–C2	2.23(1)	2.26(1)	2.26(1)
Li2–C5	2.31(1)	2.28(1)	2.29(1)
Li2–C6	2.31(1)	2.30(1)	2.34(1)
Li3–C3	2.25(1)	2.21(1)	2.23(1)
Li3–C1	2.37(1)	2.44(1)	2.63(1)
C1–C7–C8–C4	–5.1(7)	3.2(7)	9.3(7)
C2–C9–C10–C5	–30.6(7)	–34.3(9)	–29.7(8)
C3–C11–C12–C6	29.1(7)	32.2(8)	31.4(7)

We previously performed DFT calculation on Ir analog of **1** (**D**_**Ir**_ in Scheme 1) to reveal the strong donation from Ir–C σ-bonding orbital to the contacted Li^+^ ion, suggesting the formation of 3c–2e bonds among Ir–C_*ipso*_–Li.^15^ In this case, the sp^2^-hybridized lone pair on C_*ipso*_ is mainly bound to the Ir atom, resulting in an Ir–C_*ipso*_–Li bridging structure where the aryl group and the Ir atom are nearly coplanar, which can be classified as an asymmetric 3c–2e bond (Figure 2a), proposed by van Koten et al.^19^

**Figure 2:**
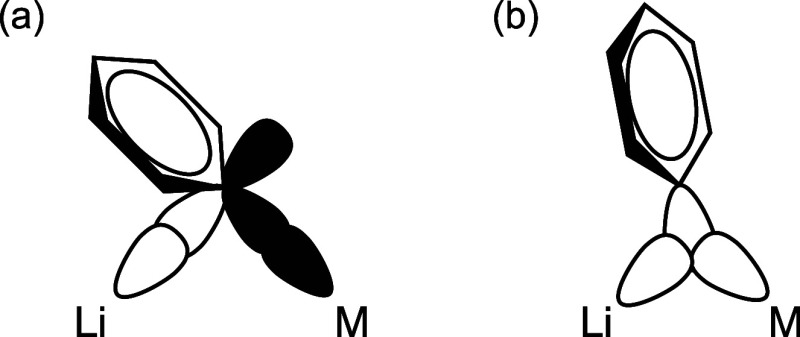
Two types for the three-center two-electron bonding mode proposed by van Koten et al.^19^ (a) Asymmetric form where aryl sp^2^ lone pair is almost exclusively bonded to M and p-orbital participates the interaction with Li. (b) Symmetric form where aryl sp^2^ lone pair interacts with both M and Li. Reproduced from ref 19. Copyright 1984 American Chemical Society.

Complex **1** is also considered to form 3c–2e bonds among Y–C_*ipso*_–Li, accounting for the elongation of Y–C bonds in **1** by coordination of Li^+^. The exceptionally long Li3–C1 bond in **1**_**C**_ can be attributed to the enhanced 3c–2e interaction in Y–C1–Li1, which is consistent with the relatively short Li1–C1 bond in **1**_**C**_ compared to **1**_**A**_ and **1**_**B**_. Since Y (1.22: Pauling) is more electropositive than Ir (2.20) and rather close to Li (0.98), complex **1** prefers rather symmetric 3c–2e bonding character, where the aryl sp^2^ lone pairs interact with both Y and Li (Figure 2b), leading to the twisted geometry of the biphenyldiyl moieties in **1.**

The molecular structure of complexes **2** and **3** was determined by single-crystal X-ray crystallography as well, showing the resemblance of these two complexes, which have three binaphthyldiyl moieties fused at the metal center (Figure 3). The crystal structure of complex **2** is described in trigonal space group *R*–3c, and the molecule has a *D*_3_-symmetric axis and displays trigonal antiprismatic geometry. This arrangement consists of two parallel equilateral triangles formed by *ipso*-carbon atoms, with a trigonal twist angle^20^ of 45.6°. Although the crystal structure of **3** shows lower symmetry compared to **2**, complex **3** also has an approximately *D*_3_-symmetric axis and shows similar trigonal antiprismatic geometry consisting of two *quasi*-equilateral triangles (side length: 3.583(8)–3.813(8) Å) facing almost parallel with a dihedral angle (C1C3C5–C2C4C6) of 2.3° (Figure S8).

**Figure 3:**
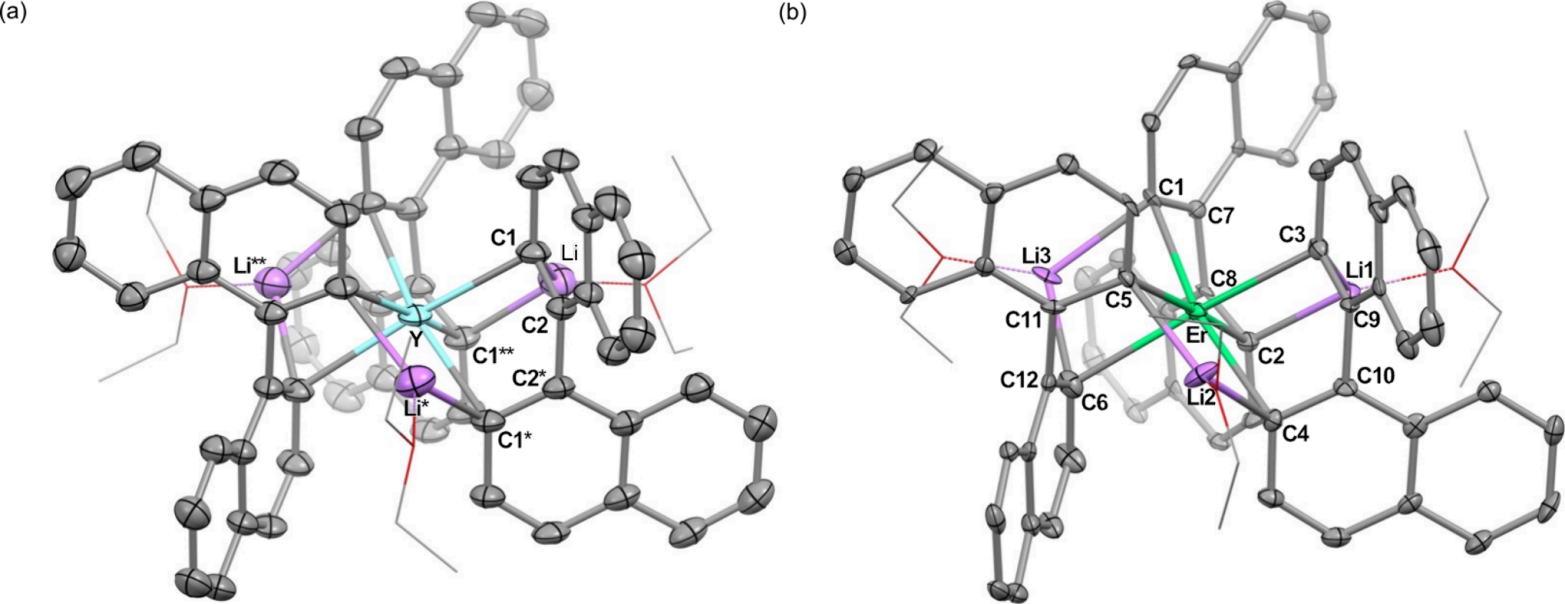
ORTEP drawing of (a) **2** and (b) **3** with thermal ellipsoids at the 50% probability level, except for coordinating Et_2_O molecules, which are shown in the wireframe model. All hydrogen atoms, noncoordinating solvent molecules, and disordered molecules are omitted for clarity.

The six naphthyl skeletons comprising the complexes have a nearly planar framework with a mean-plane deviation in the range of 0.018 Å (**2**), and 0.007–0.033 Å (**3**), which is in sharp contrast to the helical structure of naphthyl skeletons in previously reported Rh and Ir analogs (**E**_**Rh**_ and **E**_**Ir**_ in Scheme 1).^15^ Since the axial chirality of the three binaphthyl skeletons is consistent within each molecule of **2** and **3**, complexes **2** and **3** exhibit axial chirality as a whole molecule, having a mirror isomer. The obtained crystals of **2** (space group: *R*–3*c*) and **3** (space group: *P*2_1_/*c*) were racemic, each consisting of a pair of mirror isomers, forming an alternately packed structure. In both cases of complexes **2** and **3**, three Li^+^ ions contact the anionic counterpart, coordinating two *ipso*-carbon atoms on neighboring binaphthyl moieties. Besides, the deviation of Y or Er from the naphthyl planes indicates the 3c–2e bonding nature among the rare-earth metal, *ipso*-carbon, and lithium. The four-membered ring structure involving the rare-earth metal (M), one of the Li atoms and two *ipso*-carbons bridging these metal atoms features M–C distance of 2.523(2) Å for **2** and 2.485(6)–2.528(6) Å for **3**, Li–C distance of 2.223(4) Å for **2** and 2.19(1)–2.26(1) Å for **3**, C–M–C angle of 94.68(9)° for **2** and 94.1(2)–95.6(2)° for **3**, C–Li–C angle of 113.1(3)° for **2** and 110.9(5)–113.5(5)° for **3**, and M–C–Li angle of 76.10(13)° for **2** and 75.6(3)–77.2(3)° for **3** (Table 2 and Figure S8). The geometry of Er complex **3** is comparable to a hexamethyl analog, [Li­(tmeda)]_3_[Er­(CH_3_)_6_], having similar bond lengths (Er–C 2.57(2) Å, Li–C 2.22(4) Å) and angles (C–Er–C 93(1)°, C–Li–C 114(2)°, Er–C–Li 77(2)°), as reported by Schumann et al.^10^

**Table 2:** Selected Bond Lengths (Å) and Angles (deg) of Tris­(binaphthyldiyl) Metal Complexes

**2**		**3**	
Y–C1	2.523(2)	Er–C1	2.485(6)
		Er–C2	2.498(6)
		Er–C3	2.522(5)
		Er–C4	2.528(6)
		Er–C5	2.505(6)
		Er–C6	2.522(5)
Li–C1	2.223(4)	Li1–C2	2.23(1)
		Li1–C3	2.26(1)
		Li2–C4	2.22(1)
		Li2–C5	2.19(1)
		Li3–C6	2.25(1)
		Li3–C1	2.251(9)
C1*–C1**	3.717(5)	C1–C3	3.718(8)
		C3–C5	3.583(8)
		C5–C1	3.750(8)
		C2–C4	3.609(8)
		C4–C6	3.745(8)
		C6–C2	3.813(8)
C1–Y–C1**	94.68(9)	C2–Er–C3	95.5(2)
		C4–Er–C5	94.1(2)
		C6–Er–C1	95.6(2)
C1–Li–C1**	113.1(3)	C2–Li1–C3	111.9(5)
		C4–Li2–C5	113.5(5)
		C6–Li3–C1	110.9(5)
Y–C1–Li	76.10(13)	Er–C2–Li1	76.7(3)
		Er–C3–Li1	75.6(3)
		Er–C4–Li2	75.7(3)
		Er–C5–Li2	76.7(3)
		Er–C6–Li3	76.4(3)
		Er–C1–Li3	77.2(3)
C1–C2–C2*–C1*	–70.8(3)	C1–C7–C8–C2	–69.3(7)
		C3–C9–C10–C4	–64.3(7)
		C5–C11–C12–C6	–66.6(7)

### Magnetic Properties

The static and dynamic magnetic properties of **3** were investigated for a recrystallized solid-state sample under a small amount of mother liquor. Immersion of the sample in diethyl ether prevented crystal desolvation but limited the available temperature range up to 150 K due to the melting point of diethyl ether at 157 K. The temperature dependence of the product of magnetic susceptibility and temperature (χ_M_ *T*(*T*); Figure 4) is typical for complexes based on magnetically anisotropic lanthanide ions such as Er­(III). The χ_M_ *T* product decreases with decreasing temperature due to the thermal depopulation of the *m*_J_ states (Figure S12). Magnetic field dependence of magnetization (*M*(*H*)) is presented in Figure 4 (inset). The magnetization value at 7 T is 4.65 μ_B_ in comparison with the calculated value of 4.58 μ_B_. Both the χ_M_ *T*(*T*) and *M*(*H*) dependences are in very good agreement with the *ab initio* theoretical calculations using the CASSCF/SO-RASSI/SINGLE_ANISO method (Tables S4–S6 and Figures S12 and S13).

**Figure 4:**
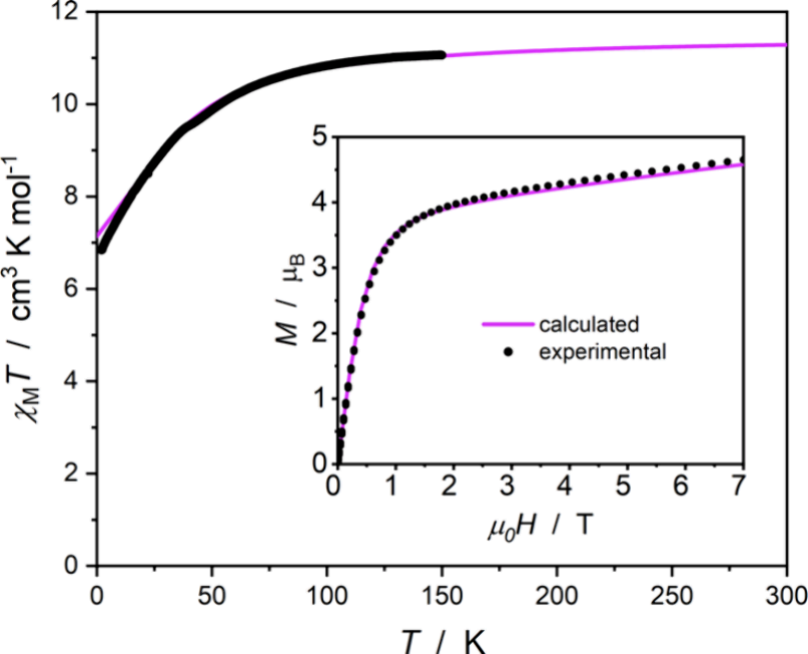
Temperature dependence of the χ_M_ *T* product for compound **3** in an applied magnetic field of 0.100 T in the 2.0–150 K range. The magnetic field dependence of the molar magnetization at 1.8 K in the 0–7 T range (inset). Black points represent experimental data, while the violet lines are obtained from the *ab initio* theoretical calculations.

The dynamic magnetic properties of **3** were studied using AC magnetic susceptibility measurements in the 10–10000 Hz frequency (ν) range. Complex **3** shows a slow magnetic relaxation only under a small applied external DC magnetic field, which allows to quench quantum tunnelling of magnetization (Figure S10, Table S2). *H*_DC_ = 0.050 T was established to be optimal for temperature dependence of χ′, χ″(ν) measurements (Figure S11, Table S3). The generalized Debye model was used to fit all AC magnetic susceptibility data (χ′, χ″(ν)) and to extract the relaxation times τ. The field dependence of τ (Figure 5) was fit using eq 1:

$$\tau^{-1}\left(H\right)=A_{1}/\left(1+A_{2}H^{2}\right)+A_{3}H^{4}+A_{4}$$ 1

where the first part describes quantum tunneling of magnetization (QTM) contribution (*A*_1_ = 5295(564) s^–1^, *A*_2_ = 1.36(22) × 10^4^ T^–2^), the second one stands for the direct relaxation process for Kramers’ ions (*A*_3_ = 1.67(5) × 10^6^ s^–1^ T^–4^) and the constant value *A*_4_ related to the contribution of the field-independent processes, Orbach and Raman (*A*_4_ = 1121(17) s^–1^). Adjusted *R* ^2^ for the fit is 0.98776.

**Figure 5:**
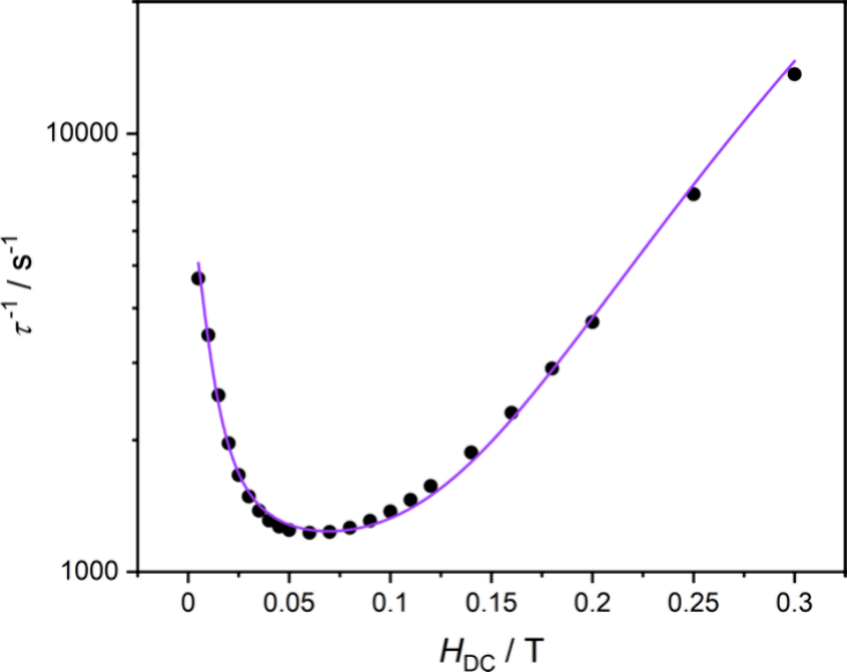
Field dependence of τ^–1^ for **3** at 1.8 K. Violet solid line is the best fit to eq 1.

The relaxation time τ extracted from the temperature dependence of the χ′, χ″(ν) were fitted using direct and Raman processes (eq 2), Figure 6:

$$\tau^{-1}\left(T\right)=C_{1}T+C_{2}T^{n}$$ 2

with fitting parameters: *C*_1_ = 452(24) s^–1^K^–1^ (direct process), *C*_2_ = 16(2) s^–1^K^–^^n^, and *n* = 5.77(7) (Raman process), *R* ^2^ = 0.99872. The Orbach process was excluded from the fitting of the temperature dependence because it led to a very low energy barrier *U*_eff_ = 13(2) cm^–1^, which is inconsistent with the energy of the first excited state (Kramers’ Doublet; KD) of 70.85 cm^–1^ obtained from the *ab initio* calculations (Table S4).

**Figure 6:**
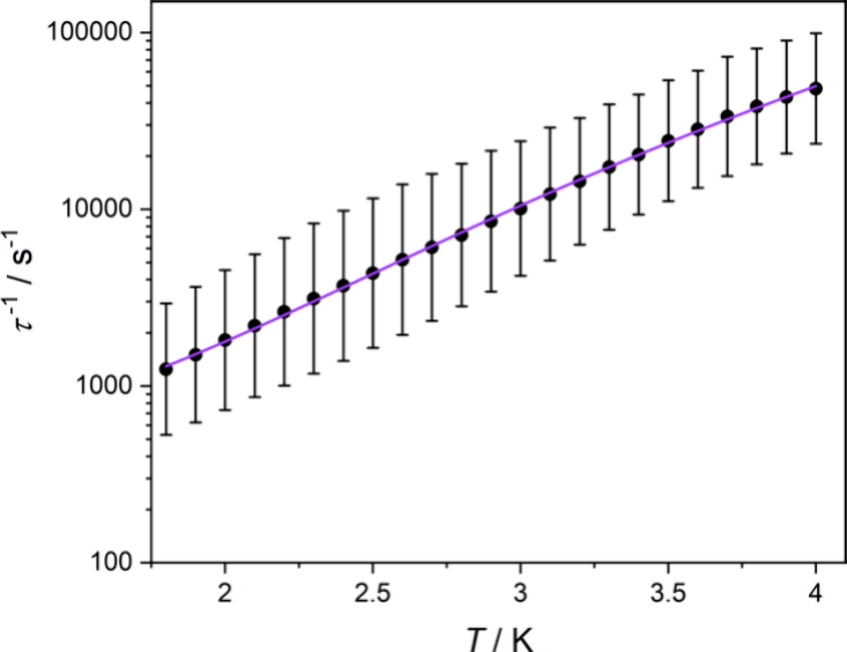
Temperature dependence of τ^–1^ for **3** in *H*_DC_ = 0.05 T. Uncertainty estimates for τ^–1^(T) were calculated based on the method of Reta and Chilton.^21^ Violet solid line is the best fit to eq 2, taking into account the calculated uncertainties.

Calculations also confirm that the ground state doublet exhibits axial character (*g*_*z*_ = 15.081, *g*_*x*_ = 0.715, *g*_*y*_ = 0.748), while the first excited state doublet at 70.85 cm^–1^ shows a much smaller *g*_*z*_ (8.757) and significantly larger *g*_*x*_ (1.411) and *g*_*y*_ (4.107), indicating a lack of axiality in this state. Although relaxation is, in principle, expected to occur via a thermally activated Orbach process involving the first excited KD, fitting of the experimental data indicates that the relaxation proceeds predominantly through a Raman process with negligible involvement of the excited KDs.

## Conclusions

We synthesized the first homoleptic rare-earth metal complexes having only three identical bidentate σ-hydrocarbyl ligands: tris­(biphenyl-2,2′-diyl)­yttrium­(III) complex **1**, and the π-extended analogs, tris­(1,1′-binaphthyl-2,2′-diyl)­yttrium­(III) complex **2**, and tris­(1,1′-binaphthyl-2,2′-diyl)­erbium­(III) complex **3**. Single-crystal X-ray crystallography revealed the trigonal prismatic geometry of **1**, along with the trigonal antiprismatic geometry of **2** and **3**, each constructed through three-centered two-electron bonds among rare-earth metal, *ipso*-carbon, and lithium. The striking contrast with the structural motif of previously reported group 9 congeners provides intriguing insights into the design of σ-hydrocarbyl metal complexes. The investigation of the static and dynamic magnetic properties of **3** revealed a slow magnetic relaxation typical of single-molecule magnets. Fitting of the magnetic behavior of **3** and the *ab initio* theoretical calculations indicated that the magnetic relaxation occurs primarily through a Raman relaxation process. Complex **3** is a rare example of an axially chiral single-molecule magnet, making it an attractive candidate for further investigation of chiral magnetic properties in enantiopure form. This motif of a rare-earth metal complex holds promise for magneto-optical applications related to information storage and processing at the molecular level.

## Experimental Section

### General Methods and Materials

Nuclear magnetic resonance spectra were recorded on a Bruker Ascend 500 spectrometer (^1^H: 500 MHz, ^13^C: 126 MHz) or a JEOL ECS400 spectrometer (^1^H: 400 MHz, ^7^Li: 194 MHz) in 5 mm NMR tubes. Chemical shift values for protons were referenced to the residual proton resonance of diethyl ether (δ: 1.06), benzene-*d*_6_ (δ: 7.16), chloroform-*d* (CDCl_3_, δ: 7.26), and dimethyl sulfoxide-*d*_6_ (DMSO-*d*_6_, δ: 2.50), respectively. Chemical shift values for ^13^C nuclei were referenced to the carbon resonance of diethyl ether (δ: 15.20). Chemical shift values for ^7^Li nuclei were reported in parts per million (ppm) relative to the external standard of LiCl in THF at δ 0.00. Elemental analyses were performed by the One-Stop Sharing Facility Center for Future Drug Discoveries, Faculty of Pharmaceutical Sciences, the University of Tokyo. Single-crystal X-ray diffraction analyses were performed on a Rigaku Varimax-dual with a Hybrid Photon Counting Detector and on a Bruker D8 Quest Eco three-circle diffractometer equipped with a Photon50 CMOS detector. The radiation was performed with Mo Kα (λ = 0.71073 Å). Powder X-ray diffraction analyses were performed by using a Bruker D8 Advance Eco diffractometer (Cu Kα radiation and graphite monochromator).

Direct current (DC) magnetic measurements were performed using a Quantum Design MPMS-3 Evercool magnetometer equipped with a 7 T magnet. Alternating current (AC) magnetic measurements were performed with a Physical Property Measurement System (PPMS). The mass of the dry sample **3** (8.9 mg) was established after magnetic measurements and after drying of the sample under vacuum for 30 min. The mass of the sample was corrected for the presence of the diamagnetic LiCl, the byproduct of the reaction, which persists despite the recrystallization procedure. The presence of LiCl was confirmed by powder X-ray diffraction (Figure S9) and was estimated to be 1.6 mg (18% of the total mass of the sample).

Unless otherwise noted, all manipulations were carried out in a glovebox filled with argon gas or with standard Schlenk techniques under nitrogen gas purified by passing through a Nikka Seiko dry column DC-L4 (NIKKA SEIKO Co., LTD). Air- and moisture-sensitive liquids and solutions were transferred via a syringe.

Anhydrous tetrahydrofuran (THF), diethyl ether (Et_2_O), and hexane were purchased from Kanto Chemical Co., Inc. (Kanto) and purified by the method of Pangborn et al.,^22^ followed by further dryness over sodium–potassium alloy (NaK) and passed through a pad of dry silica gel before use. Anhydrous benzene, anhydrous pentane, THF-*d*_8_, and benzene-*d*_6_ were purchased from Kanto, dried over NaK, and passed through a pad of dry silica gel before use. Yttrium­(III) chloride (anhydrous, Fujifilm Wako chemicals (Wako)), erbium­(III) chloride (anhydrous, Sigma-Aldrich), 2,2′-dibromo-1,1′-biphenyl (Tokyo Chemical Industry Co., Ltd. (TCI)), 2,2′-dibromo-1,1′-binaphthyl (Wako), ^*n*^butyllithium (hexane solution, Kanto), DMSO-*d*_6_ (Kanto), chloroform-*d* (Kanto), methanol-*d*_4_ (Kanto) and dibromomethane (CH_2_Br_2_, Wako) were purchased and used as received. 2,2′-Dilithiobiphenyl,^23^ sodium 2,6-di-*tert*-butyl-4-methylphenolate (NaBHT),^24^ and Er­(BHT)_3_ ^25^ were prepared according to the literature procedures. 2,2′-Dilithio-1,1′-binaphthyl was prepared by a modification of the literature procedure.^26^ 2,2′-Dilithiobiphenyl and 2,2′-dilithio-1,1′-binaphthyl are potentially pyrophoric and should be handled under an inert atmosphere.

### 2,2′-Dilithiobiphenyl (**5**)

To a solution of 2,2′-dibromobiphenyl (2.10 g, 6.73 mmol) in THF (70 mL) was slowly added a hexane solution of ^*n*^BuLi (1.59 M, 10.0 mL) at −78 °C, and the reaction solution was stirred for 15 h to gradually give a white suspension. White precipitates were collected by filtration, and the white solid was washed with hexane under a nitrogen atmosphere to provide **5** as a white solid (1.84 g, 3.78 mmol) in 73% yield. ^1^H NMR (400 MHz, THF-*d*_8_): δ 7.95 (d, *J* = 6.4 Hz, 4H), 7.10 (dd, *J* = 7.2, 7.0 Hz, 2H), 6.66 (dd, *J* = 6.8, 6.8 Hz, 2H): ^13^C NMR (126 MHz, THF-*d*_8_): δ 174.7 (br, C_*ipso*_), 155.4, 142.8, 125.8, 123.0.

### 2,2′-Dilithio-1,1′-binaphthyl•xTHF (**6**)

In a Schlenk tube, to a solution of racemic 2,2′-dibromo-1,1′-binaphthyl (1.27 g, 3.08 mmol) in THF (15.0 mL) was slowly added a hexane solution of ^*n*^BuLi (1.52 M, 4.2 mL) at −78 °C, and the reaction mixture was stirred for 1 h, followed by a slow addition of hexane (60 mL) to gradually give pale yellow suspension. After gradually warming the reactor to room temperature, the yellow precipitate was collected by filtration and dried under reduced pressure in a glovebox to provide racemic **6** as a pale yellow solid (57.7 wt % purity, 1.42 g, 3.08 mmol). This procedure afforded **6** in 53.5–69.1 wt % purity. The solubility of **6** was low in THF, making characterization by NMR analysis difficult. The purity of **6** was determined by quenching it with a small amount of methanol-*d*_4_ to convert it to 1,1′-binaphthyl-2,2′-*d*_2_, drying under reduced pressure, and then subjecting the resulting compound to NMR measurements in the presence of CH_2_Br_2_ as an internal standard (Figure S3).

### [Li­(thf)_2_]_3_[Y­(biphenyl-2,2′-diyl)_3_] (**1**)

In a glovebox, YCl_3_ (41.2 mg, 0.211 mmol) and 2,2′-dilithiobiphenyl (118.3 mg, 0.712 mmol) were dissolved in THF (1.0 mL) and stirred for 10 min at room temperature. To the resulting solution was added hexane (10 mL) to afford a suspension. The precipitate was removed by filtration. The remaining solution was concentrated under reduced pressure, gradually forming colorless needle-like crystals. When the volume of the mother liquor was reduced to ca. 1 mL, the crystals that formed were collected. The obtained crystals were washed with a small portion of hexane, and further dryness under reduced pressure afforded complex **1** as colorless powder (24.4 mg, 12% yield). ^1^H NMR (500 MHz, Et_2_O): δ 7.68 (d, *J* = 7.0 Hz, 6H), 7.58 (d, *J* = 8.0 Hz, 6H), 7.04 (dd, *J* = 7.5, 7.5 Hz, 6H), 6.84 (dd, *J* = 7.0, 7.0 Hz, 6H). ^13^C­{^1^H} NMR (126 MHz, Et_2_O): δ 185.72 (d, ^1^ *J*_Y–C_ = 23.7 Hz, C_*ipso*_), 158.30, 141.49, 127.85, 124.95, 123.47. ^7^Li NMR (194 MHz, Et_2_O): δ –0.02. Elemental analysis: calcd (%) for C_60_H_72_Li_3_O_6_Y: C 72.14, H 7.27; found: C 72.34, H 6.98.

### [Li­(Et_2_O)]_3_[Y­(1,1′-binaphthyl-2,2′-diyl)_3_] (**2**)

In a glovebox, YCl_3_ (41.2 mg, 0.211 mmol) and 2,2′-dilithio-1,1′-binaphthyl•xTHF (53.5 wt % purity, 340.7 mg, 0.685 mmol) were dissolved in THF (10 mL) and left at room temperature without stirring. Complex **2** was precipitated in the resulting mixture within 62 h. After the brown supernatant was removed, the precipitate was washed with Et_2_O and dried under reduced pressure to give the crude product as a colorless powder (83.4 mg). Following recrystallization, employing slow diffusion of pentane into a benzene solution afforded complex **2** as colorless crystals (61.1 mg, 27% yield). Elemental analysis: calcd (%) for C_72_H_66_Li_3_O_3_Y: C 79.41, H 6.11; found: C 79.41, H 6.26.

### [Li­(Et_2_O)]_3_[Er­(1,1′-binaphthyl-2,2′-diyl)_3_] (**3**)

In a glovebox, ErCl_3_ (109.0 mg, 0.40 mmol) and 2,2′-dilithio-1,1′-binaphthyl•xTHF (69.1 wt % purity, 616.0 mg, 1.60 mmol) were suspended in THF (20 mL) and stirred at room temperature for 1 h. The resulting solution was evaporated to dryness and extracted with Et_2_O. Following recrystallization from slowly evaporating Et_2_O afforded complex **3** as small colorless crystals in a dark brown solution. The colorless crystals were filtered and washed two times with a small amount of fresh Et_2_O. As both the product and LiCl (byproduct) are soluble in Et_2_O, both were present after recrystallization, with the total mass being 86.8 mg. No other products were found in the recrystallized mixture, as confirmed by powder X-ray diffraction, which was compared with the diffractogram generated from the structure models obtained from single-crystal X-ray crystallography. Based on the DC magnetic measurements, the amount of LiCl in the bulk sample was determined to be 18 wt %; therefore, the amount of **3** in the bulk sample was estimated to be 71.2 mg (15% yield). The same product can be obtained by starting from Er­(BHT)_3_ (BHT = 2,6-di-*tert*-butyl-4-methylphenolate) instead of ErCl_3_.

### *Ab* *Initio* Calculations

OpenMolcas (ver. 24.10) was used for *ab initio* single-point calculations with the CASSCF/SO-RASSI/SINGLE_ANISO approach.^27^ Atomic coordinates were sourced from a single-crystal X-ray diffraction experiment of [Li­(Et_2_O)]_3_[Er­(1,1′-binaphthyl-2,2′-diyl)_3_] (**3**) without a crystallization solvent molecule. The active space of the Complete Active Space Self-Consistent-Field method comprised 11 electrons in seven 4f-type orbitals (CASSCF­(11,7)). State average calculations on all 35 quartet and 112 doublet spin states were conducted, and the resulting spin states were mixed by spin–orbit coupling within the restricted active space state interaction (SO-RASSI) approach. Scalar-relativistic Douglas-Kroll-Hess Hamiltonian with relativistic ANO-RCC basis sets was used.^28^ Basis set: Er.ANO-RCC-VDZP, C.ANO-RCC-VDZP for coordinating carbon atoms and ANO-RCC-VDZ for remaining C, O, Li, and H atoms was used. Calculation of two-electron integrals utilized Cholesky decomposition with a 1.0 × 10^–8^ threshold to save disk space. Based on the RASSI calculated states, local magnetic properties were calculated with the SINGLE_ANISO module. Energies of the lowest lying multiplets Kramers' Doublets, pseudospin g-tensors, crystal field parameters, and other additional data are presented in Tables S4–S6 and Figures S12 and S13.

## Supplementary Material



## Notes

Spectroscopic data for novel compounds, details for X-ray diffraction analyses, magnetic measurements, and theoretical calculations (PDF)

## References

[1] Desgranges A., D'Agosto F., Boisson C. (2024). Rare-Earth Metallocenes for Polymerization
of Olefins and Conjugated Dienes: From Fundamental Studies to Olefin
Block Copolymers. ChemPlusChem.

[2] Zimmermann M., Anwander R. (2010). Homoleptic Rare-Earth Metal Complexes
Containing Ln–C
σ-Bonds. Chem. Rev..

[3] Schumann, H. , Genthe, W. Organometallic Compounds of the Rare Earths. In Handbook on the Physics and Chemistry of Rare Earths, Vol. 7; Elsevier, 1984; pp 445–571.

[4] Wilkinson G., Birmingham J. M. (1954). Cyclopentadienyl Compounds of Sc, Y, La, Ce and Some
Lanthanide Elements. J. Am. Chem. Soc..

[5] Arndt S., Okuda J. (2002). Mono­(cyclopentadienyl)
Complexes of the Rare-Earth Metals. Chem. Rev..

[6] Hart F. A., Saran M. S. (1968). Triphenylscandium
and Related Compounds. Chem. Commun. (London).

[7] Collier M. R., Lappert M. F., Truelock M. M. (1970). μ-Methylene
Transition Metal Binuclear Compounds: Complexes with Me_3_SiCH_2_– and Related Ligands. J. Organomet. Chem..

[8] Yagupsky G., Mowat W., Shortland A., Wilkinson G. (1970). Trimethylsilylmethyl Compounds of Transition Metals. J. Chem. Soc. D.

[9] Evans W. J., Shreeve J. L., Broomhall-Dillard R.
N. R., Ziller J. W. (1995). Isolation
and Structure of a Homoleptic Yttrium Trimethylsilylmethyl Complex. J. Organomet. Chem..

[10] Schumann H., Mueller J., Bruncks N., Lauke H., Pickardt J., Schwarz H., Eckart K. (1984). Organometallic Compounds
of the Lanthanides.
17. Tris­[(tetramethylethylenediamine)­lithium] Hexamethyl Derivatives
of the Rare Earths. Organometallics.

[11] Wayda A. L., Evans W. J. (1978). Synthesis and Thermal
Decomposition of Homoleptic *tert*-Butyl Lanthanide
Complexes. J.
Am. Chem. Soc..

[12] Harder S. (2005). Syntheses
and Structures of Homoleptic Lanthanide Complexes with Chelating *o*-Dimethylaminobenzyl Ligands: Key Precursors in Lanthanide
Chemistry. Organometallics.

[13] Pandey P., Yu X., Panetti G. B., Lapsheva E., Gau M. R., Carroll P. J., Autschbach J., Schelter E. J. (2023). Synthesis, Electrochemical, and Computational
Studies of Organocerium­(III) Complexes with Ce–Aryl Sigma Bonds. Organometallics.

[14] Wittig G., Rümpler K. D. (1971). Über
die Reaktionsweise von 2.2’-Dilithium-biphenyl gegenüber
Metallhalogeniden, III. Die atropisomeren *o*-Hexaphenylene. Liebigs Ann. Chem..

[15] Hara M., Hirooka Y., Iwasaki T., Nozaki K. (2024). Synthesis, Structure,
and Optical Property of Tris­(biaryldiyl)metal Complexes Consisting
of Group 9 Elements. Inorg. Chem..

[16] Woodruff D.
N., Winpenny R. E., Layfield R. A. (2013). Lanthanide
Single-Molecule Magnets. Chem. Rev..

[17] Arndt S., Okuda J. (2005). Cationic Alkyl Complexes of the Rare-Earth Metals: Synthesis, Structure,
and Reactivity. Adv. Synth. Catal..

[18] Arndt S., Spaniol T. P., Okuda J. (2003). Homogeneous
Ethylene-Polymerization
Catalysts Based on Alkyl Cations of the Rare-Earth Metals: Are Dicationic
Mono­(alkyl) Complexes the Active Species?. Angew.
Chem., Int. Ed..

[19] van
Koten G., Jastrzebski J. T. B.
H., Stam C. H., Niemann N. C. (1984). Solid-State Structure of [Au_2_Li_2_(C_6_H_4_CH_2_NMe_2_-2)_4_]): A Model for the Reactive Structure of Organocuprates. J. Am. Chem. Soc..

[20] McCusker J. K., Rheingold A. L., Hendrickson D. N. (1996). Variable-Temperature Studies of Laser-Initiated ^5^T_2_ → ^1^A_1_ Intersystem
Crossing in Spin-Crossover Complexes: Empirical Correlations between
Activation Parameters and Ligand Structure in a Series of Polypyridyl
Ferrous Complexes. Inorg. Chem..

[21] Reta D., Chilton N. F. (2019). Uncertainty Estimates
for Magnetic Relaxation Times
and Magnetic Relaxation Parameters. Phys. Chem.
Chem. Phys..

[22] Pangborn A.
B., Giardello M. A., Grubbs R. H., Rosen R. K., Timmers F. J. (1996). Safe and
Convenient Procedure for Solvent Purification. Organometallics.

[23] Hübner A., Diehl A. M., Bolte M., Lerner H.-W., Wagner M. (2013). High-Temperature
Reactivity of the Strongly Electrophilic Pristine 9*H*-9-Borafluorene. Organometallics.

[24] Semeniuchenko V., Braje W. M., Organ M. G. (2021). Sodium
Butylated Hydroxytoluene:
A Functional Group Tolerant, Eco-Friendly Base for Solvent-Free, Pd-Catalysed
Amination. Chem. – Eur. J..

[25] Zhang H., Nakanishi R., Katoh K., Breedlove B. K., Kitagawa Y., Yamashita M. (2018). Low Coordinated Mononuclear Erbium­(III)
Single-Molecule Magnets with *C*
_3*v*
_ Symmetry: A Method for Altering Single-Molecule Magnet Properties
by Incorporating Hard and Soft Donors. Dalton
Trans..

[26] Buchalski P., Pacholski R., Shkurenko A., Suwińska K. (2015). Novel, Axially
Chiral Analogues of Nickelocene with Nickeladibenzofluorenyl Ligand. J. Organomet. Chem..

[27] Malmqvist P.
Å., Roos B. O., Schimmelpfennig B. (2002). The Restricted
Active Space (RAS) State Interaction Approach with Spin–Orbit
Coupling. Chem. Phys. Lett..

[28] Douglas M., Kroll N. M. (1974). Quantum Electrodynamical
Corrections to the Fine Structure of Helium. Ann. Phys..

