# Supplementary material for: Synthesis, Structure, and Property of Tris(biphenyldiyl)yttrium(III) Tris(binaphthyldiyl)yttrium(III) and Tris(binaphthyldiyl)erbium(III) Complexes
Source: Inorg Chem. 2025 Jun 16;64(25):12540–7. doi: 10.1021/acs.inorgchem.5c00532 (PMC12216231; doi:10.1021/acs.inorgchem.5c00532)
Supplement: Supplementary file 1 [file ic5c00532_si_001.pdf]

Supporting information for

Synthesis, Structure, and Property of Tris(biphenyldiyl)yttrium(III), Tris(binaphthyldiyl)yttrium(III), and Tris(binaphthyldiyl)erbium(III) Complexes

Masaki Hara,† Gabriela Handzlik,‡ Mirosław Arczyński,‡ Takanori Iwasaki,† Dawid Pinkowicz,‡ Kyoko Nozaki†

†Department of Chemistry and Biotechnology, Graduate School of Engineering, The University of Tokyo, 7-3-1 Hongo, Bunkyo-ku 113-8656 Tokyo, Japan.

‡Faculty of Chemistry, Jagiellonian University, Gronostajowa 2, 30-387 Kraków, Poland.

E-mail: iwasaki@chembio.t.u-tokyo.ac.jp; dawid.pinkowicz@uj.edu.pl

Table of Contents

1. NMR Data S2

2. X-ray Diffraction Data S6

3. Magnetic Properties S10

4. Calculations S14

5. References S17

1. NMR Data

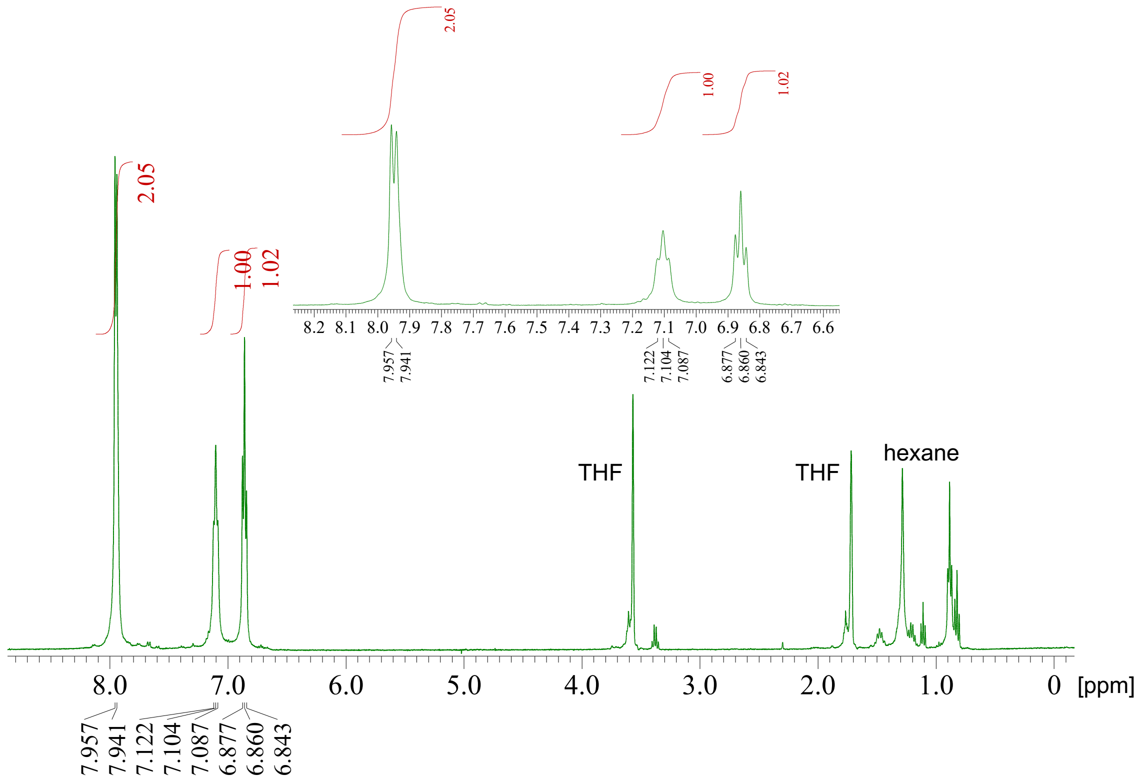

Figure S1. ^{1}H NMR (400 MHz, THF-d_8) spectrum of 2,2'-dilithiobiphenyl (**5**).

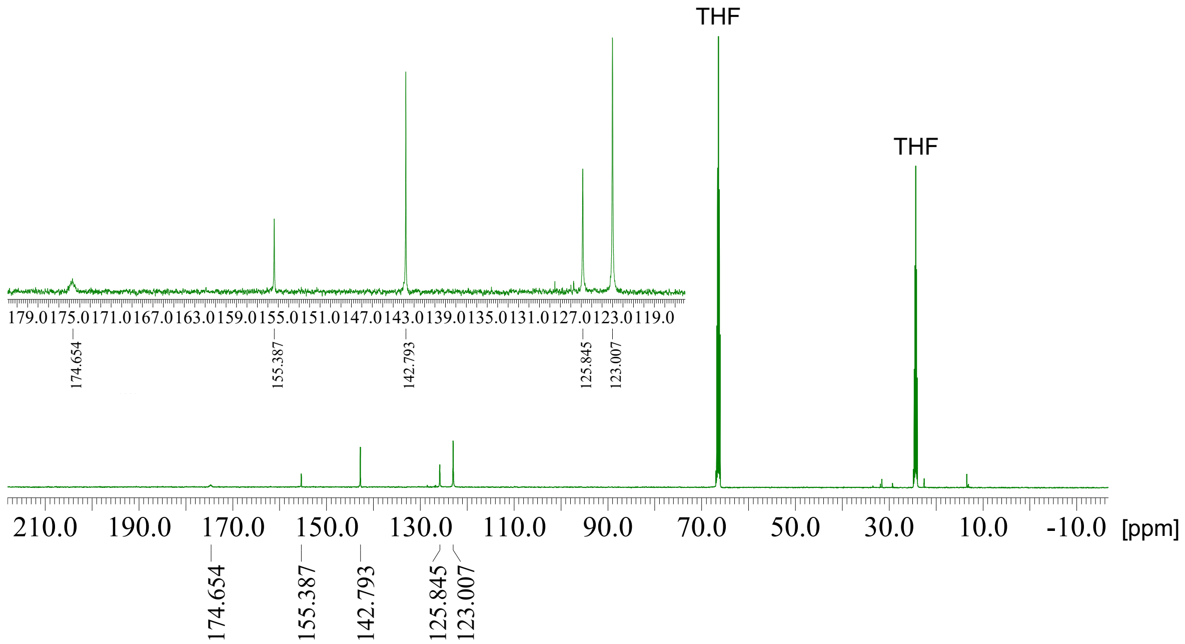

Figure S2. $^{13}C\{^{1}H\}$ NMR (126 MHz, THF-d_8) spectrum of 2,2'-dilithiobiphenyl (**5**).

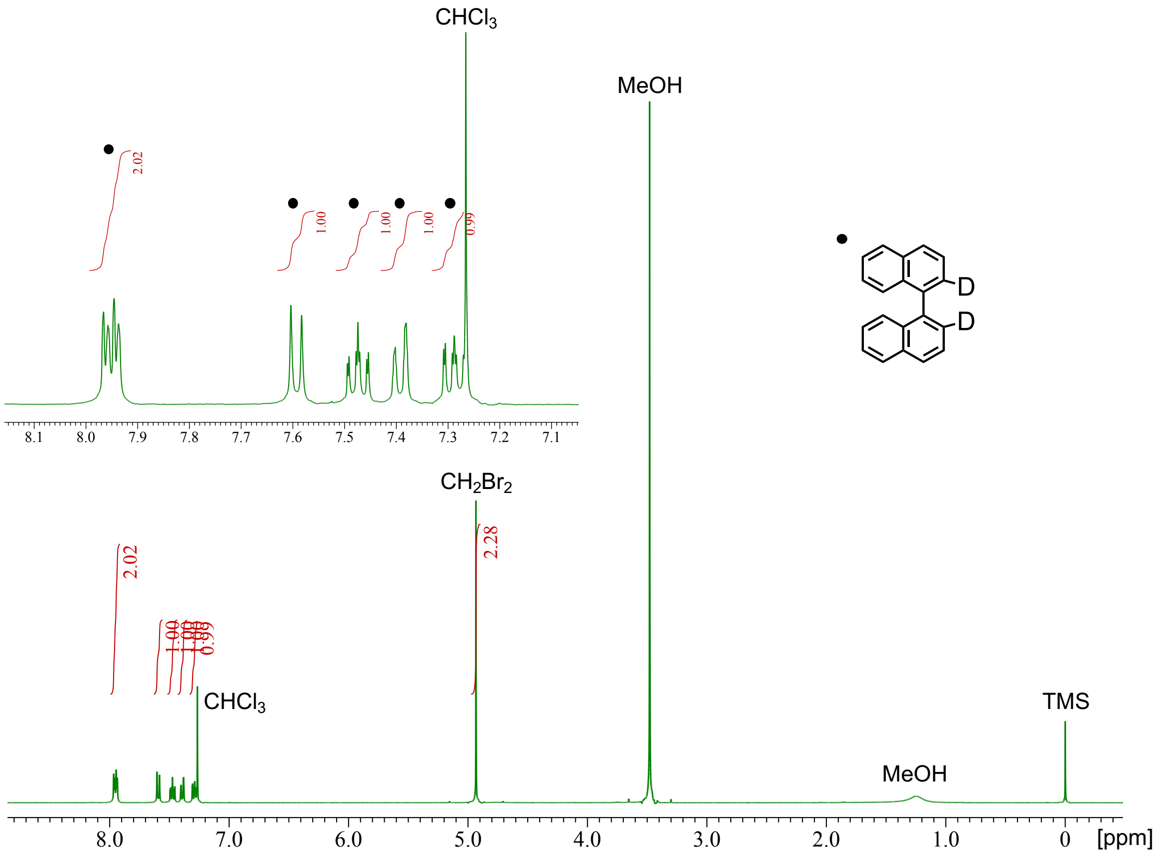

Figure S3. ^{1}H NMR spectrum (400 MHz, $CDCl_3$) of 1,1'-binaphthyl-2,2'-d_2 obtained by quenching 2,2'-dilithio-1,1'-binaphthyl•xTHF (**6**) with methanol-d_4. In this sample, 12.8 mg of 2,2'-dilithio-1,1'-binaphthyl•xTHF was quenched by a small amount of methanol-d_4, dried under reduced pressure, and then subjected to NMR measurements in the presence of 11.0 mg of CH_2Br_2 as an internal standard, determining its purity of 57.7 wt %. Chemical shift values were referenced to the residual proton resonance of $CDCl_3$ (δ: 7.26).

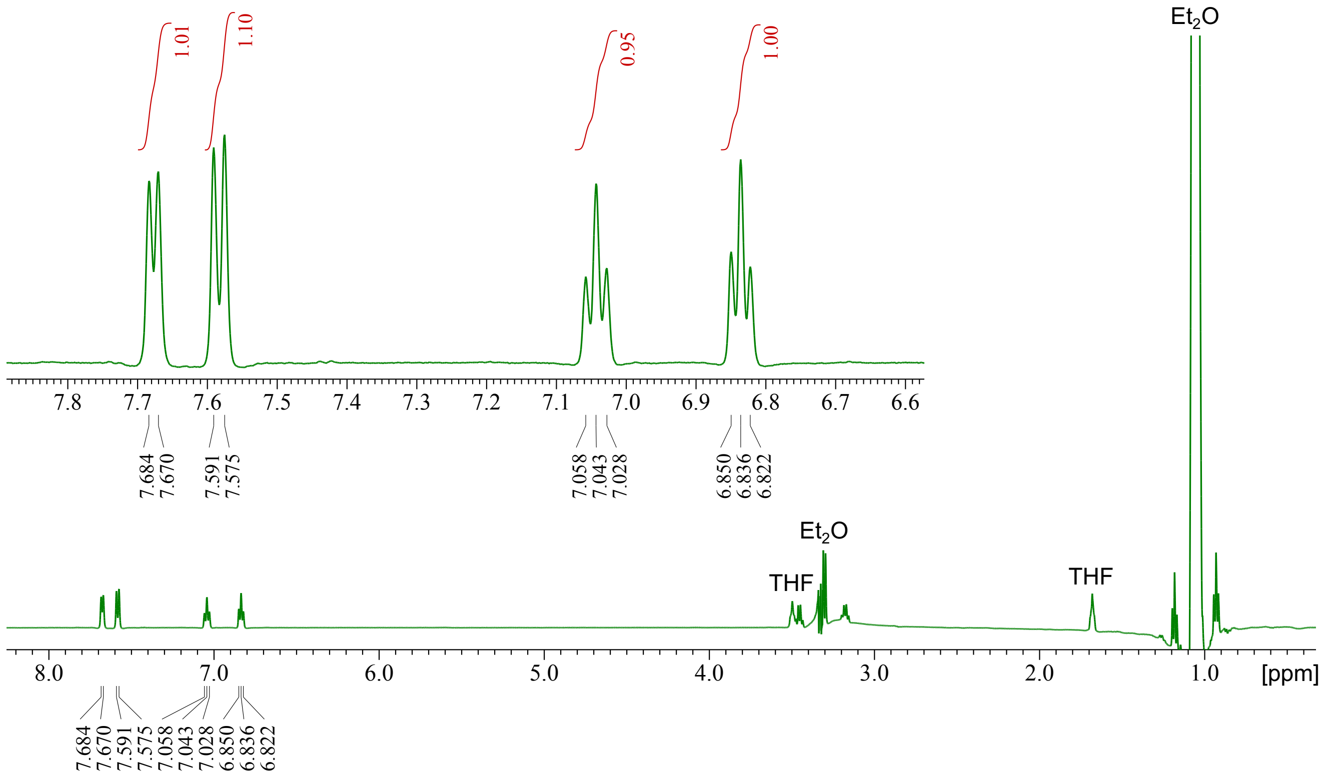

Figure S4. ^{1}H NMR (500 MHz, Et_2O) spectrum of $[Li(thf)_2]_3[Y(biphenyl\text{-}2,2'\text{-}diyl)_3]$ (**1**).

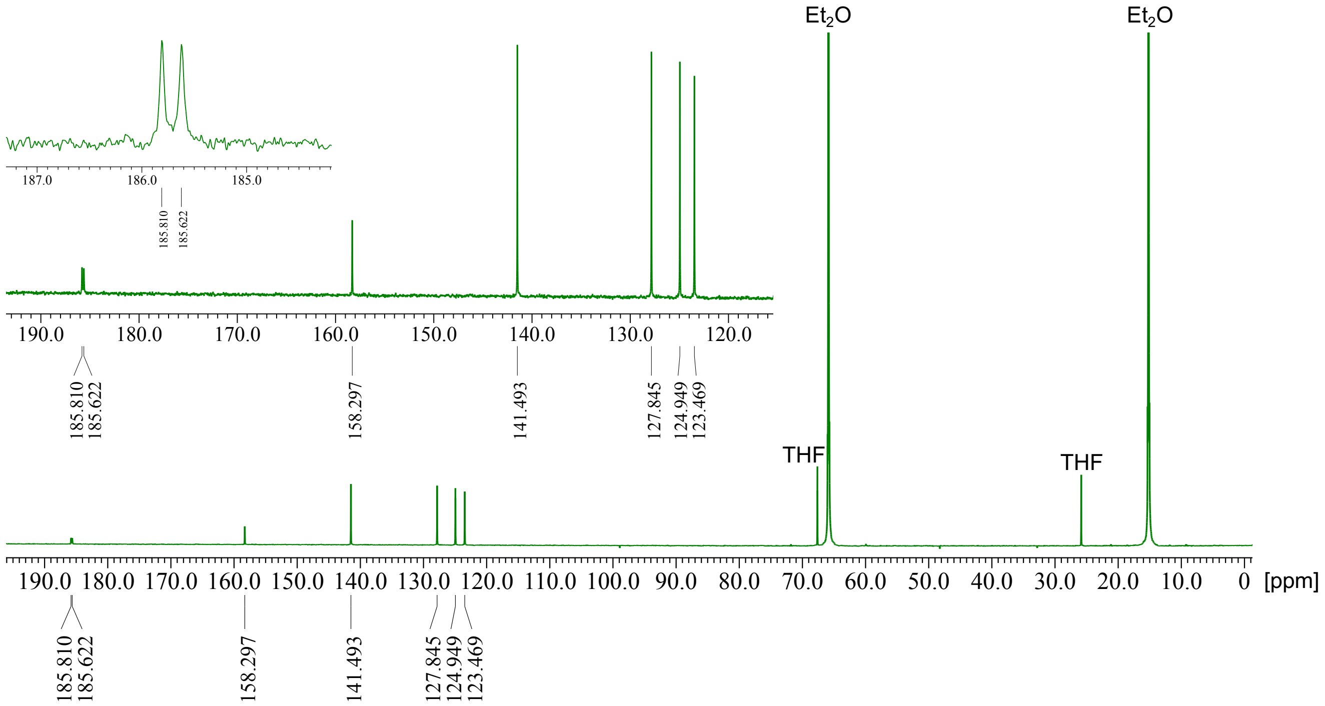

Figure S5. ^{13}C{^{1}H} NMR (126 MHz, Et_2O) spectrum of $[Li(thf)_2]_3[Y(biphenyl\text{-}2,2'\text{-}diyl)_3]$ (**1**).

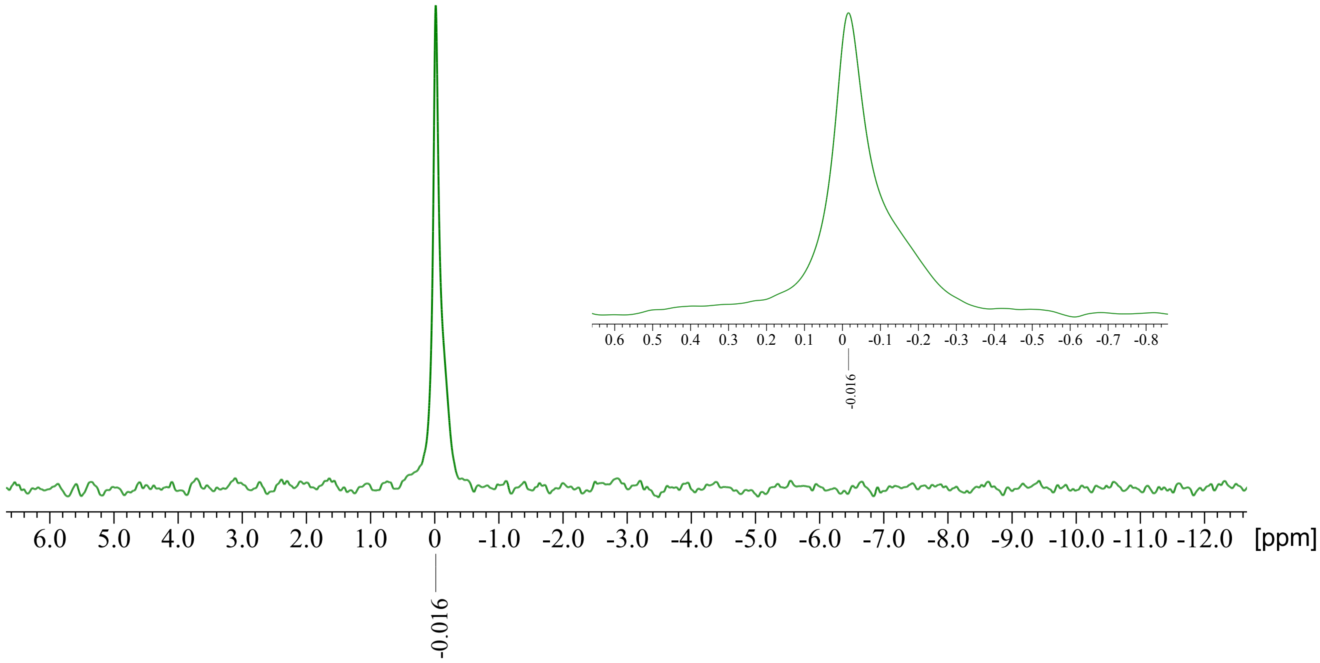

Figure S6. ^{7}Li NMR (194 MHz, Et_2O) spectrum of $[Li(thf)_2]_3[Y(biphenyl\text{-}2,2'\text{-}diyl)_3]$ (**1**).

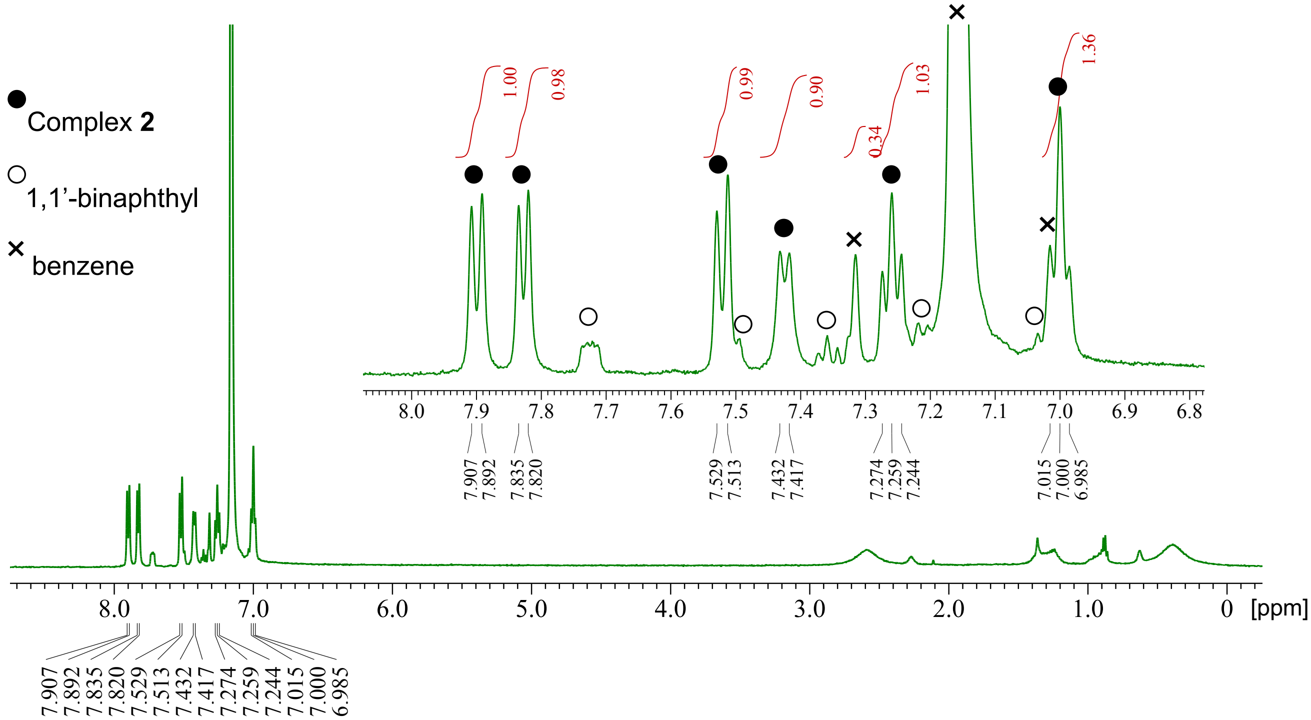

Figure S7. ^{1}H NMR (500 MHz, C_6D_6) spectrum of $[Li(Et_2O)]_3[Y(1,1'\text{-}binaphthyl\text{-}2,2'\text{-}diyl)_3]$ (**2**). It should be noted that contamination of 1,1'-binaphthyl via the partial decomposition of **2** was observed.

2. X-ray Diffraction Data

Details of the single crystal X-ray diffraction (scXRD) data are listed in **Table S1**. The scXRD analyses for $[Li(thf)_2]_3[Y(biphenyl-2,2'-diyl)_3]$ (**1**) and $[Li(Et_2O)]_3[Y(1,1'-binaphthyl-2,2'-diyl)_3]$ (**2**) were performed on a Rigaku VariMax-dual with Hybrid Photon Counting Detector. The radiation was performed with graphite monochromated Mo Kα (λ = 0.71073 Å) at 50 kV and 24 mA. The scXRD analyses for $[Li(Et_2O)]_3[Er(1,1'-binaphthyl-2,2'-diyl)_3]$ (**3**) were collected on a Bruker D8 Quest Eco three-circle diffractometer equipped with a Photon50 CMOS detector, a Mo Kα sealed tube X-ray radiation source and a Triumph monochromator. All the following procedures for analysis, Olex2[S1] was used as a graphical interface. The structure was solved by direct methods with SHELXT[S2] and refined by full-matrix least-squares techniques against F^2 with SHELXL.[S3] The intensities were corrected for Lorentz and polarization effects. The non-hydrogen atoms were refined anisotropically. Hydrogen atoms were placed using AFIX instructions. The MERCURY program[S4] was used to draw the molecular structures.

The purity of the bulk sample of **3** was confirmed by powder X-ray diffraction (PXRD; Figure S9). PXRD experiments were carried out using Bruker D8 Advance Eco diffractometer (Cu Kα radiation source and graphite monochromator) at room temperature for samples loaded into a narrow borosilicate-glass capillaries (0.7 mm in diameter) with small amount of mother solutions. Strong signals around 2θ = 30° and 35° registered for sample obtained just after synthesis comes from LiCl which is a byproduct of the reaction.[S5] After recrystallization, these two signals disappear almost completely, and crystallinity of the sample is significantly improved (amorphous hump disappeared). Very good agreement of the reflections on experimental and simulated PXRD diffraction patterns confirms the identity and purity the sample used for magnetic measurements. Small amount of diamagnetic LiCl in the sample after recrystallization does not affect magnetic properties of the sample.

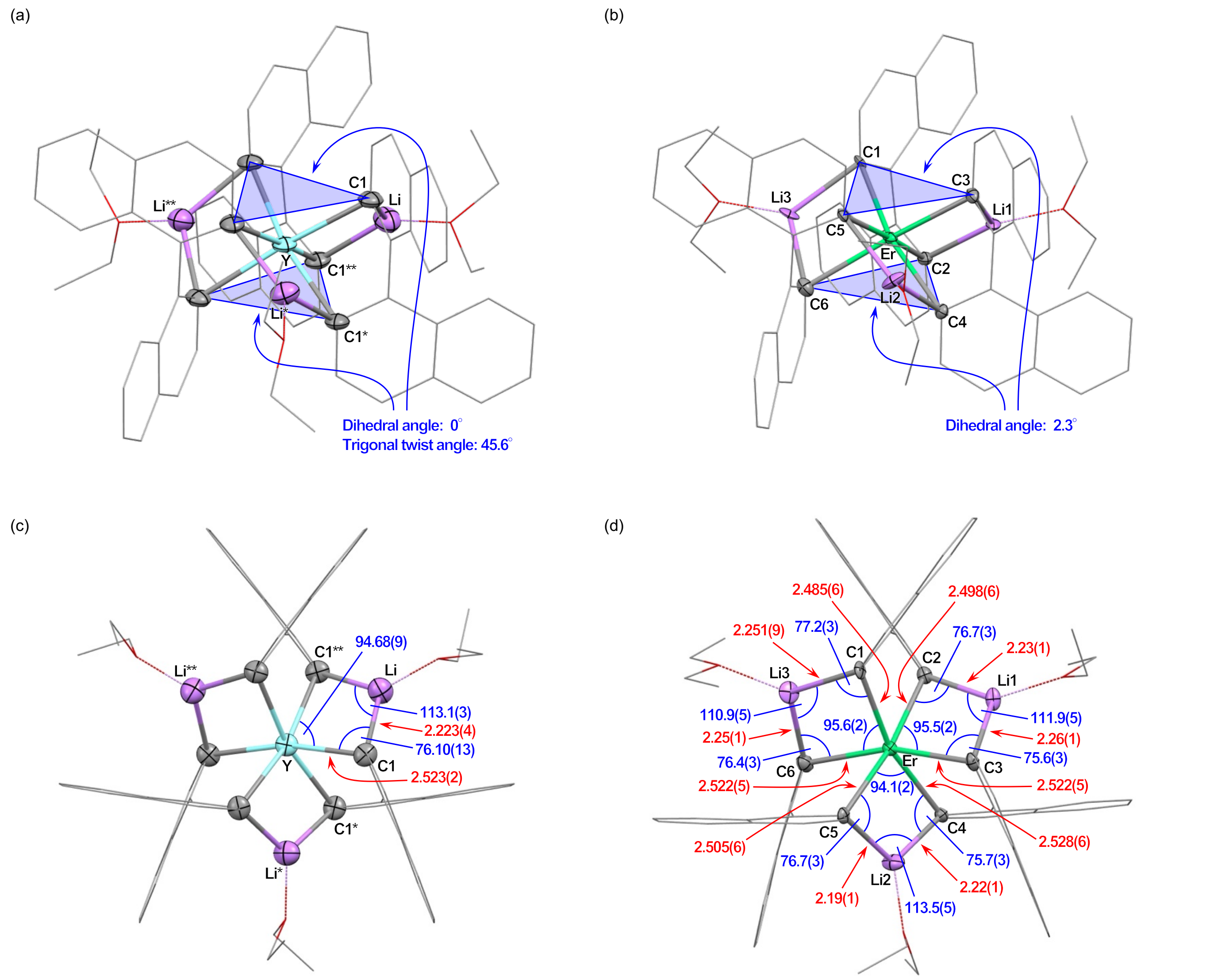

Figure S8. ORTEP drawing of **2** and **3** with thermal ellipsoids at the 50% probability level, except for binaphthyl skeletons and coordinating Et_2O molecules, which are shown in the wireframe model. All hydrogen atoms, non-coordinating solvent molecules, and disordered molecules are omitted for clarity. (a) describes trigonal antiprismatic geometry of **2**, consisting of two parallel equilateral triangles formed by *ipso*-carbon atoms, with a trigonal twist angle of 45.6°. (b) describes trigonal antiprismatic geometry of **3**, consisting of two *quasi*-equilateral triangles of *ipso*-carbon atoms aligned almost parallel with a dihedral angle of 2.3°. (c) describes bond lengths (red, Å) and angles (blue, deg) in Y complex **2**. (d) describes bond lengths (red, Å) and angles (blue, deg) in Er complex **3**.

Table S1. Crystallographic data and structure refinement details of complexes **1**, **2** and **3**.

		**1**	**2**	**3**
CCDC number		2391819	2391818	2418906
Molecular formula		$C_{180}H_{216}Li_9O_{18}Y_3$	$C_{78}H_{72}Li_3O_3Y$	$C_{148}H_{142}Er_2Li_6O_7$
Formula weight		2996.71	1167.08	2408.77
Temperature (K)		93	93	100(2)
Crystal system		triclinic	trigonal	monoclinic
Space group		*P*–1	*R*–3*c*	$P2_1/c$
Unit cell dimensions	*a* (Å)	10.5211(4)	14.2023(5)	20.8819(10)
	b (Å)	24.3166(11)	14.2023(5)	14.3791(7)
	c (Å)	32.3997(16)	56.7617(14)	20.5071(9)
	α (°)	76.025(4)	90	90
	β (°)	82.828(4)	90	104.934(2)
	γ (°)	78.449(4)	120	90
Volume (Å^3)		7855.3(6)	9915.2(7)	5949.5(5)
Z		2	6	2
Density (calculated) (g/cm^3)		1.267	1.173	1.345
μ (mm^{-1})		1.164	0.929	1.46
F(000)		3168	3672	2480
Crystal size (mm^3)		0.05 × 0.05 × 0.05	0.2 × 0.2 × 0.2	0.17 × 0.15 × 0.11
Radiation		Mo K*α* (λ = 0.71073)	Mo K*α* (λ = 0.71073)	Mo K*α* (λ = 0.71073)
2Θ range for data collection (°)		4.404 to 57.19	6.128 to 59.184	4.928 to 54.206
Index ranges		$-13 \leq h \leq 14$	$-19 \leq h \leq 18$	$-26 \leq h \leq 26$
		$-32 \leq k \leq 32$	$-19 \leq k \leq 19$	$-17 \leq k \leq 18$
		$-42 \leq l \leq 43$	$-70 \leq l \leq 78$	$-26 \leq l \leq 26$
Reflections collected		113704	41925	82824
Independent reflections		35990	2958	13126
	R_{int}	0.1534	0.0537	0.0706
	R_{sigma}	0.2657	0.0260	0.0498
Data/restraints/parameters		35990/0/1765	2958/0/165	13126/81/765
Goodness-of-fit on F^2		1.016	1.059	1.193
Final R indexes [I ≥ 2σ (I)]	R_1	0.1145	0.0464	0.0676
	wR_2	0.1887	0.1251	0.1172
Final R indexes [all data]	R_1	0.2353	0.0645	0.0810
	wR_2	0.2310	0.1378	0.1217
Largest diff. peak/hole (e Å^{-3})		1.39/–1.34	0.58/–0.56	2.27/–4.21

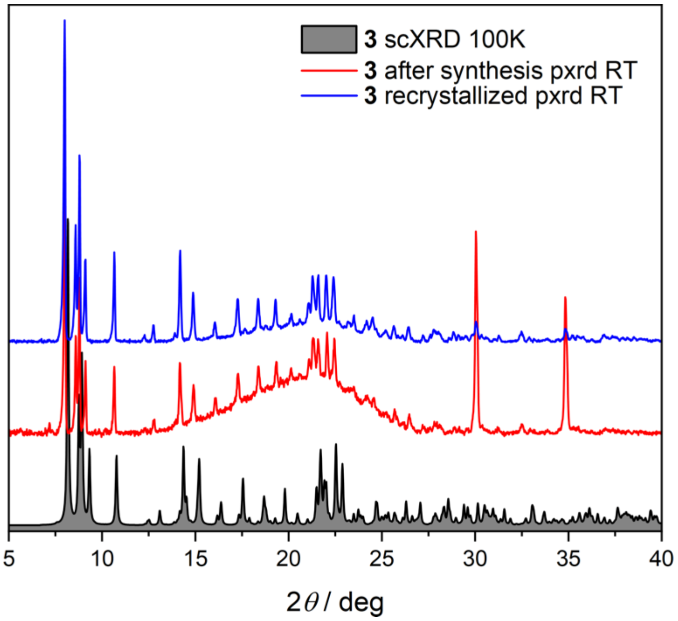

Figure S9. Comparison of experimental powder X-ray diffractograms of **3** before (red line) and after recrystallization (blue line) and diffractogram generated for the crystal structure determined for monocrystal of **3** (black line). Both experimental powder X-ray diffractograms were collected at room temperature.

3. Magnetic Properties

Alternating current (AC) magnetic properties

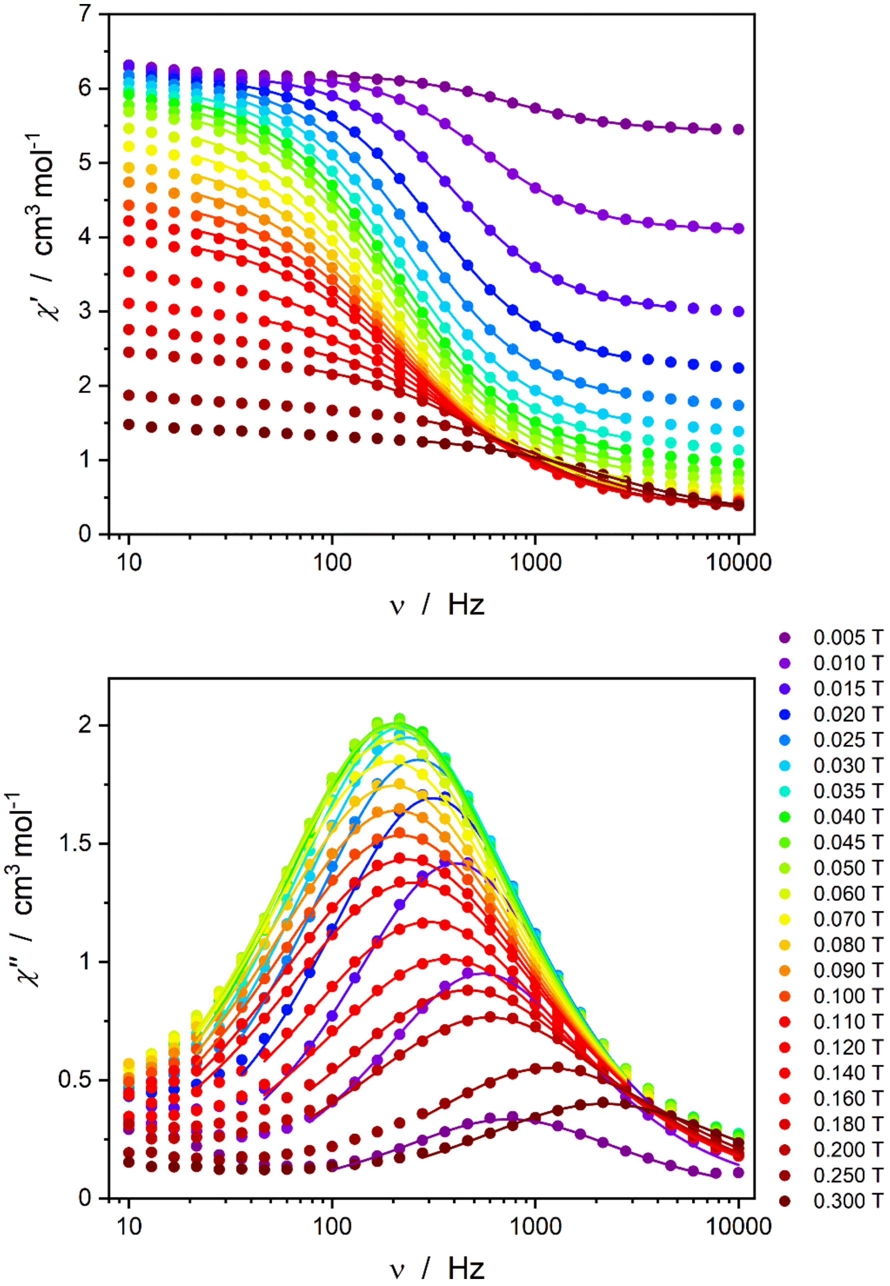

Figure S10. In-phase (χ') and out-of-phase (χ'') AC susceptibilities for **3** at 1.8 K measured in various fields H_{DC}. Values of α and τ parameters are presented in Table S2.

Table S2. Values of α and τ from fitting one modified Debye function to $\tau(\nu)$ dependencies for **3** at 1.8 K.

H (T)	α	τ (s)
0.050	0.110(8)	2.14(3) × 10^{-4}
0.010	0.073(5)	2.88(2) × 10^{-4}
0.015	0.084(4)	3.95(2) × 10^{-4}
0.020	0.100(4)	5.09(44) × 10^{-4}
0.025	0.115(4)	6.01(3) × 10^{-4}
0.030	0.127(3)	6.73(4) × 10^{-4}
0.035	0.141(4)	7.25(5) × 10^{-4}
0.040	0.149(4)	7.63(5) × 10^{-4}
0.045	0.157(4)	7.88(5) × 10^{-4}
0.050	0.163(3)	8.02(5) × 10^{-4}
0.060	0.172(3)	8.14(5) × 10^{-4}
0.070	0.181(3)	8.10(5) × 10^{-4}
0.080	0.191(3)	7.93(5) × 10^{-4}
0.090	0.198(3)	7.64(5) × 10^{-4}
0.100	0.205(4)	7.28(5) × 10^{-4}
0.110	0.212(4)	6.85(5) × 10^{-4}
0.120	0.217(5)	6.37(6) × 10^{-4}
0.140	0.200(2)	5.34(3) × 10^{-4}
0.160	0.198(3)	4.33(3) × 10^{-4}
0.180	0.201(3)	3.43(2) × 10^{-4}
0.200	0.200(5)	2.69(2) × 10^{-4}
0.250	0.176(5)	1.37(1) × 10^{-4}
0.300	0.195(8)	0.73(1) × 10^{-4}

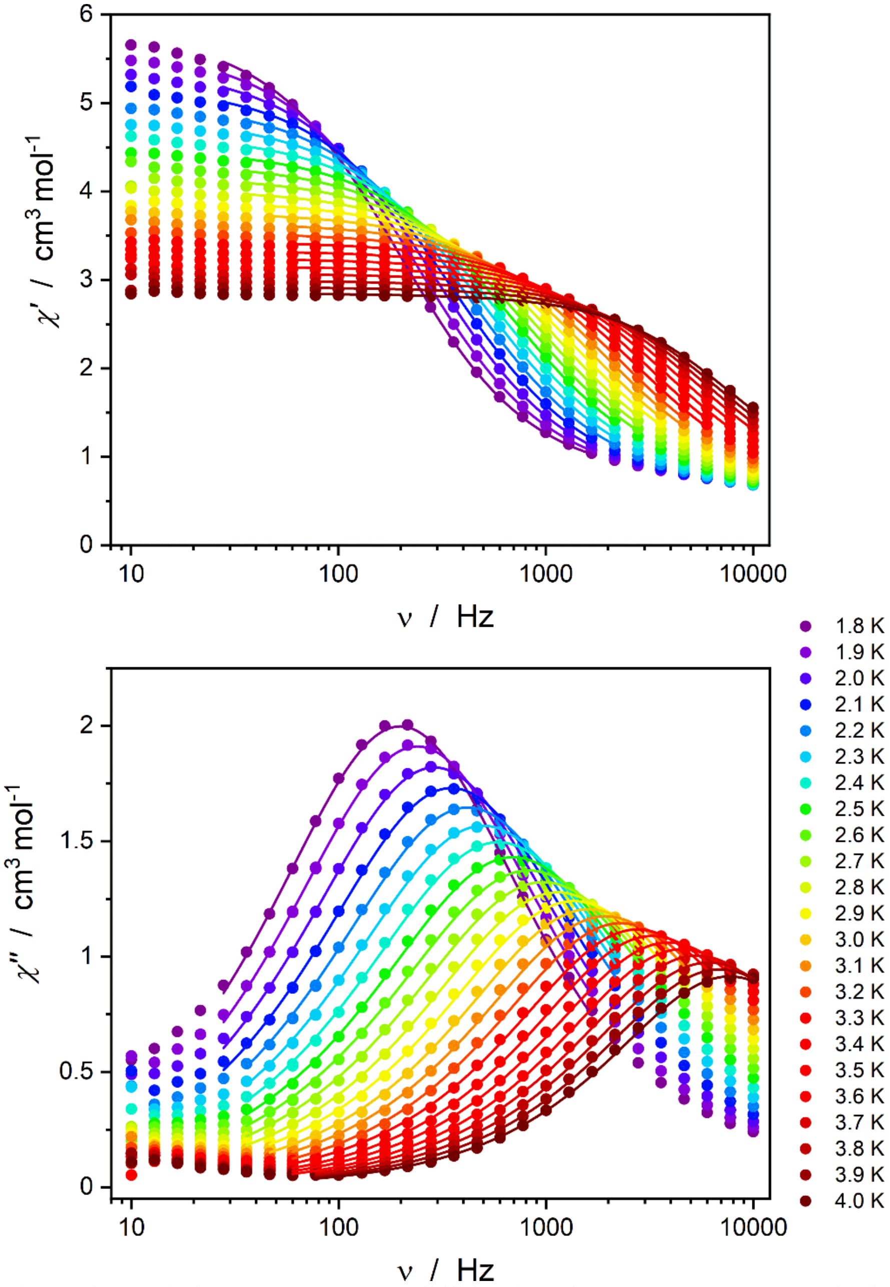

Figure S11. In-phase (χ') and out-of-phase (χ'') AC susceptibilities for **3** in a 0.050 T DC magnetic field. Values of α and τ parameters are presented in Table S3.

Table S3. Values of α and τ from fitting one modified Debye function to $\tau(\nu)$ dependencies for **3** in H_{DC} = 0.050 T.

T (K)	α	τ (s)
1.8	0.157(3)	$8.04(4) \times 10^{-4}$
1.9	0.165(3)	$6.65(3) \times 10^{-4}$
2.0	0.172(3)	$5.49(3) \times 10^{-4}$
2.1	0.177(4)	$4.56(3) \times 10^{-4}$
2.2	0.185(4)	$3.80(3) \times 10^{-4}$
2.3	0.190(4)	$3.20(3) \times 10^{-4}$
2.4	0.190(5)	$2.71(3) \times 10^{-4}$
2.5	0.188(5)	$2.30(3) \times 10^{-4}$
2.6	0.190(6)	$1.93(2) \times 10^{-4}$
2.7	0.184(6)	$1.64(2) \times 10^{-4}$
2.8	0.177(6)	$1.40(2) \times 10^{-4}$
2.9	0.173(6)	$1.17(2) \times 10^{-4}$
3.0	0.163(6)	$0.99(2) \times 10^{-4}$
3.1	0.160(6)	$0.82(1) \times 10^{-4}$
3.2	0.149(6)	$0.69(1) \times 10^{-4}$
3.3	0.147(6)	$5.77(9) \times 10^{-5}$
3.4	0.137(6)	$4.90(8) \times 10^{-5}$
3.5	0.139(6)	$4.09(6) \times 10^{-5}$
3.6	0.133(6)	$3.53(6) \times 10^{-5}$
3.7	0.136(6)	$2.98(5) \times 10^{-5}$
3.8	0.130(6)	$2.61(4) \times 10^{-5}$
3.9	0.125(6)	$3.32(4) \times 10^{-5}$
4.0	0.121(6)	$2.07(4) \times 10^{-5}$

4. Calculations

Table S4. Pseudospin Hamiltonian g tensors and energy of the ground $^4I_{15/2}$ Er multiplets Kramers' Dublets (KD).

KD	g_z	g_y	g_x	E (cm^{-1})
1	15.081	0.748	0.715	0
2	8.757	4.107	1.411	70.847
3	10.310	4.044	0.850	74.540
4	10.285	3.030	0.596	103.976
5	5.912	2.691	1.037	114.409
6	9.742	4.313	3.273	293.206
7	0.086	5.820	7.004	325.068
8	10.429	4.923	2.497	348.673

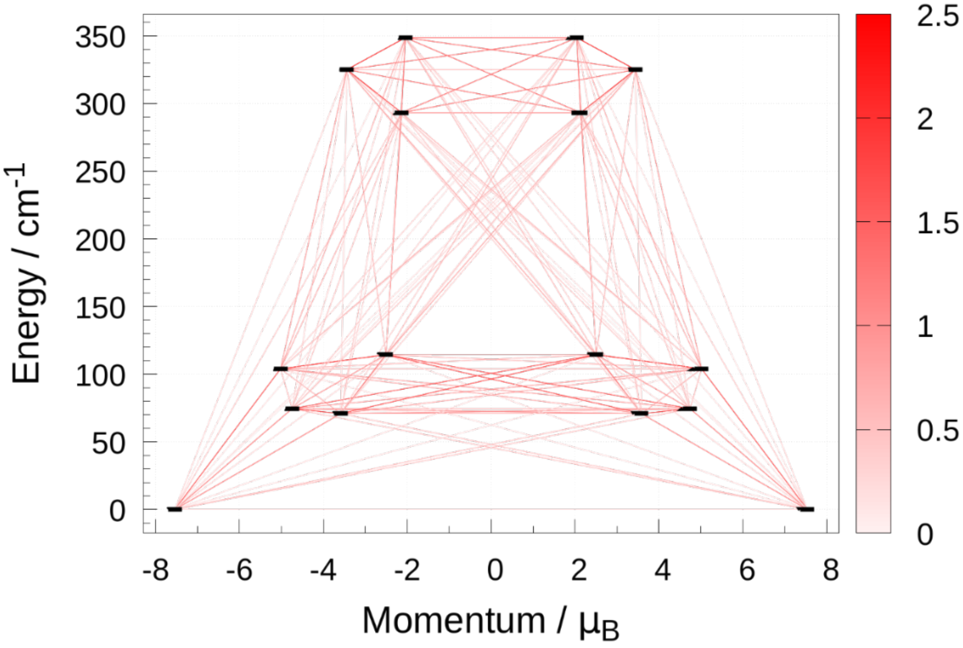

Figure S12. Graphical representation of the lowest energy structure of **3** based on ab initio calculations. Red connecting lines show transitions between states and their color's intensity indicates qualitatively the relative probability of such transitions: darker shade means higher chance. Following the darkest red lines potential relaxation of magnetization pathways in **3** can be assessed.

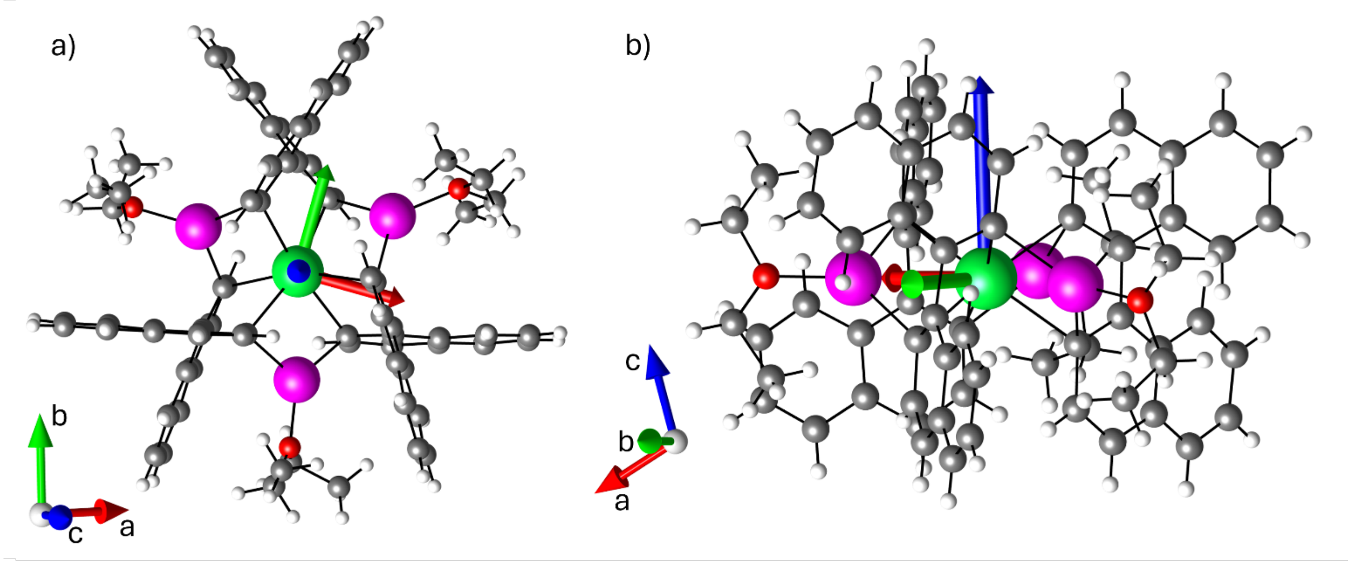

Figure S13. Calculated *ab initio* (details in text) magnetic axes of the lowest Kramers' Doublet of **3** in two different orientations of coordinate system. Main magnetic axis is highlighted by being longer than others, but the length of each axis is not proportional to related g factors (compare Table S4). Color scheme: "z" magnetic axis is shown in blue, "x" is red and "y" green, Er green, C grey, O red, Li purple, H white. Figure was made with help of the Vesta program.[S6]

Table S5. Decomposition of the ground $^4I_{15/2}$ Er multiplet into the wave functions with definite projection of the total moment on the quantization axis (values in %).

	Ab initio state no.															
|m_J>	1	2	3	4	5	6	7	8	9	10	11	12	13	14	15	16
|−15/2>	0	2	31.1	8.4	33.5	21.2	0.3	0	3.1	0.2	0.1	0	0.2	0	0	0
|−13/2>	0.6	92.5	1.5	0.8	0.6	0.3	0.4	0.9	0.2	0	1	0.2	0	0.1	1	0
|−11/2>	0.2	1.2	3.9	0.5	3.3	1.1	29.6	35.9	2.7	0.7	13.2	4.7	0.9	0.8	0.1	1.2
|−9/2>	0	0	0.1	0.1	0.2	0.3	3.4	0.1	19.7	7.4	1.7	1.8	61.7	0.3	0	3.2
|−7/2>	0	0.7	4.3	2.9	3.4	1	1.6	1.9	1.3	0.2	13.5	2.3	0.6	2.9	62.5	0.7
|−5/2>	0	0.1	1.2	3.1	0.1	1.5	8.5	10.8	4.2	0.3	28.8	13.5	1.4	2.5	2	22
|−3/2>	0	0.6	1.7	1	1.6	1.2	4.3	0.5	57.1	0.2	0.1	1.9	19.5	6.9	0.6	2.8
|−1/2>	0	2.1	23	16.4	24.4	6.3	0.6	1.2	2.7	0.1	10.5	6.8	2.2	0	3	0.7
| 1/2>	2.1	0	16.4	23	6.3	24.4	1.2	0.6	0.1	2.7	6.8	10.5	0	2.2	0.7	3
| 3/2>	0.6	0	1	1.7	1.2	1.6	0.5	4.3	0.2	57.1	1.9	0.1	6.9	19.5	2.8	0.6
| 5/2>	0.1	0	3.1	1.2	1.5	0.1	10.8	8.5	0.3	4.2	13.5	28.8	2.5	1.4	22	2
| 7/2>	0.7	0	2.9	4.3	1	3.4	1.9	1.6	0.2	1.3	2.3	13.5	2.9	0.6	0.7	62.5
| 9/2>	0	0	0.1	0.1	0.3	0.2	0.1	3.4	7.4	19.7	1.8	1.7	0.3	61.7	3.2	0
| 11/2>	1.2	0.2	0.5	3.9	1.1	3.3	35.9	29.6	0.7	2.7	4.7	13.2	0.8	0.9	1.2	0.1
| 13/2>	92.5	0.6	0.8	1.5	0.3	0.6	0.9	0.4	0	0.2	0.2	1	0.1	0	0	1
| 15/2>	2	0	8.4	31.1	21.2	33.5	0	0.3	0.2	3.1	0	0.1	0	0.2	0	0

Table S6. Crystal field parameters B_k^q and their weights on the total crystal field splitting. Only parameters with weights higher than 1% are shown. Crystal field parameters given in accordance with ref. S7.

k	q	B_k^q	Weight (%)
**4**	–3	7.092×10^{-3}	14.37614
**6**	0	6.691×10^{-5}	12.71975
**4**	3	5.809×10^{-3}	11.77413
**6**	–6	5.945×10^{-5}	11.30074
**2**	0	–1.005	11.2208
**6**	–3	-3.186×10^{-5}	6.056027
**4**	0	-2.889×10^{-3}	5.856232
**6**	3	-2.596×10^{-5}	4.934197
**6**	1	-2.455×10^{-5}	4.667028
**6**	6	-1.310×10^{-5}	2.489459
**6**	–5	1.307×10^{-5}	2.485543
**4**	1	1.006×10^{-3}	2.038905
**4**	–4	-8.762×10^{-4}	1.776032
**4**	4	-6.544×10^{-4}	1.326458
**4**	2	6.471×10^{-4}	1.311605
**6**	–1	-5.679×10^{-6}	1.079629

5. References

(S1) Dolomanov, O. V.; Bourhis, L. J.; Gildea, R. J.; Howard, J. A. K.; Puschmann, H. OLEX2: A Complete Structure Solution, Refinement and Analysis Program. *J. Appl. Crystallogr.* **2009**, *42*, 339–341.

(S2) Sheldrick, G. M. SHELXT – Integrated Space-Group and Crystal-Structure Determination. *Acta Crystallogr., Sect. A: Found. Adv.* **2015**, *71*, 3–8.

(S3) Sheldrick, G. M. Crystal Structure Refinement with SHELXL. *Acta Crystallogr., Sect. C: Struct. Chem.* **2015**, *71*, 3–8.

(S4) Macrae, C. F.; Sovago, I.; Cottrell, S. J.; Galek, P. T. A.; McCabe, P.; Pidcock, E.; Platings, M.; Shields, G. P.; Stevens, J. S.; Towler, M.; Wood, P. A. Mercury 4.0: From Visualization to Analysis, Design and Prediction. *J. Appl. Crystallogr.* **2020**, *53*, 226–235.

(S5) Cortona, P. Direct Determination of Self-Consistent Total Energies and Charge Densities of Solids: A Study of the Cohesive Properties of the Alkali Halides. *Phys. Rev. B Condens Matter* **1992**, *46*, 2008–2014.

(S6) Momma, K.; Izumi, F. VESTA 3 for Three-Dimensional Visualization of Crystal, Volumetric and Morphology Data. *J. Appl. Crystallogr.* **2011**, *44*, 1272–1276.

(S7) Chibotaru, L. F.; Ungur, L. *Ab Initio* Calculation of Anisotropic Magnetic Properties of Complexes. I. Unique Definition of Pseudospin Hamiltonians and Their Derivation. *J. Chem. Phys.* **2012**, *137*, 064112.
